# Structural characterization of intra- and intermolecular disulfide bonds in voltage-dependent anion channel 3 (VDAC3) protein from *Rattus norvegicus* by high-resolution mass spectrometry

**DOI:** 10.1007/s00216-025-06074-w

**Published:** 2025-08-28

**Authors:** Maria Gaetana Giovanna Pittalà, Annamaria Cucina, Stefano Conti-Nibali, Vincenzo Cunsolo, Antonella Di Francesco, Giuseppe Battiato, Simona Reina, Salvatore Foti, Vito De Pinto, Rosaria Saletti

**Affiliations:** 1 Organic Mass Spectrometry Laboratory, Department of Chemical Sciences, University of Catania, Viale A. Doria 6, 95125 Catania, Italy; 2 Section of Biology and Genetics, Department of Biomedical Sciences and Biotechnology, University of Catania, Via S. Sofia, 97, 95123 Catania, Italy

**Keywords:** High-resolution mass spectrometry, Intra- and intermolecular disulfide bonds, Rattus voltage-dependent anion selective channel isoform 3 (rVDAC3), Cysteine redox state, Structural characterization

## Abstract

Voltage-dependent anion channels, the most abundant proteins of the mitochondrial outer membrane, are responsible for the exchange of ions and metabolites between cytosol and mitochondria. They participate in the control of glycolytic metabolism through interaction with numerous enzymes and play a key role in the regulation of mitochondria-mediated apoptosis, cancer, and neurodegenerative diseases. The enzymatic digestion procedure in solution, originally developed in our laboratory, followed by high-resolution mass spectrometry coupled with UHPLC, has proven to be a powerful tool for the structural characterization of these “difficult” proteins. In this work, we used this procedure for the localization of intramolecular disulfide bonds in rVDAC3 and also for the characterization of intermolecular disulfide bonds formed by this protein with other VDAC isoforms. As a result, three intramolecular and seven intermolecular disulfide bonds between rVDAC3 with rVDAC1 and rVDAC2 were uniquely characterized. Furthermore, evidence was obtained for the existence of two additional intramolecular disulfide bonds between Cys2/Cys8 with Cys36 and Cys122, although these identifications were not supported by MS/MS spectra. The formation of intermolecular disulfide bonds helps to explain the previously observed VDAC oligomerization and demonstrates that disulfide bridges are directly involved in their homo- or hetero-oligomerization. Data are available via ProteomeXchange with identifier PXD064110.

**Graphical Abstract:**

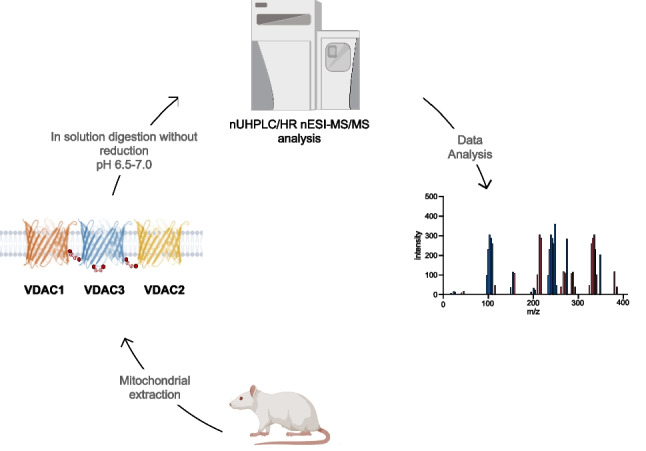

**Supplementary Information:**

The online version contains supplementary material available at 10.1007/s00216-025-06074-w.

## Introduction

Voltage-dependent anion channel isoforms (VDACs) are the most abundant membrane proteins of the mitochondrial outer membrane (OMM) [1–3]. Their main recognized role is to form pores in the OMM, as shown in [4]. New functions and roles of VDAC isoforms are gradually being discovered [5–7]. Three different VDAC isoforms, encoded by separate genes, have been characterized in higher eukaryotes, named VDAC1, VDAC2, and VDAC3, in order of their discovery [8]. The three-dimensional structures of mouse and human VDAC1 isoform were determined by X-ray crystallography and NMR [9–11]. These studies revealed that the structures for the mouse and human VDAC1 isoform consist of 19 β-strands arranged to form a trans-membrane β-barrel and a region containing an *α*-helix at the N-terminus of the protein. The barrel is organized as a regular antiparallel array of β-strands, with the exception of strands 1 and 19 that run in parallel. The available X-ray and NMR data, however, do not allow determining the exact position and local structure of the amphipathic *α*-helix tail. The structure of zebrafish VDAC2 was also solved at high resolution, confirming the same β-barrel arrangement as VDAC1 [12]. More recently, the structure of a pair of human VDAC2, as a component of a PINK1 complex with the translocase of the outer membrane, was determined at 3.1-angstrom resolution by cryo–electron microscopy [6]. The amino acid sequence of VDACs is highly conserved. The sequence identity/similarity between the human VDAC1 and VDAC3 is 75%/91%, while it is 67%/85% between VDAC2 and VDAC3 [8]. The high sequence similarity allowed the prediction of the 3-D structure of VDAC2 and VDAC3 by means of homology modeling [8, 13].

VDACs are responsible for the exchange of adenosine nucleotides, sugars, and inorganic ions between the mitochondrial and cytoplasmic compartments. They also participate in the control of glycolytic metabolism through interaction with numerous metabolic enzymes and play a key role in the regulation of mitochondria-mediated apoptosis [14]. Due to their role in cellular metabolism and apoptosis, VDACs are directly implicated in cancer and neurodegeneration regulation [15–17], and for these reasons, VDACs have attracted interest as pharmacological target [18, 19].

The structural characterization of VDAC isoforms presents challenging issues. Indeed, although VDACs are enriched in the unbound fraction eluted from hydroxyapatite (HTP) chromatography, other membrane proteins with similar hydrophobicity are also present [20]. Since there is no established protocol for the isolation of a single VDAC isoform, they can be analyzed in the mixture from HTP elution. Furthermore, VDAC3 is the least abundant isoform, tenfold less abundant than VDAC2 and 100-fold less abundant than VDAC1B [21]. This quantitative aspect adds further difficulties to the structural characterization of the VDAC3 isoform [22]. Recently, we have demonstrated that the molecular-level structural characterization of VDACs can be achieved by high-resolution mass spectrometry (HR-MS), which has the unique ability to successfully analyze complex protein mixture [20, 23–28]. Indeed, the investigation of rVDAC3 by reduction, alkylation, and a tryptic and chymotryptic digestion procedure in solution, originally developed in our laboratory [23, 25], followed by nUHPLC/HR nESI-MS/MS analysis, allowed us to distinguish the cysteines that in the native protein are in free form or engaged in disulfide bonds from those that are totally or partially in the trioxidized form.

An important goal for understanding the structure of VDAC proteins and the role of cysteine residues in VDAC functions under physiological conditions is to achieve a complete map of the localization of disulfide bonds. There are still few studies on the disulfide bonds of VDAC proteins in the literature. Moreover, in most of these studies, S–S bonds are investigated by molecular dynamic simulations or are predicted indirectly using molecular biology techniques [29].

Several VDAC3 intramolecular disulfide bonds were simulated by molecular dynamics in [30]. In this paper, Guardiani et al. hypothesized disulfide bridges formation between Cys2 or Cys8, located in the N-terminal tail, and the cysteines positioned at the bottom of the pore and exposed to the IMS (as Cys122). The structures were evaluated by measuring the reduction of the channel current flow in the presence of the predicted disulfide bridges; nevertheless, it is necessary to consider that the simulations obtained are dependent on the three-dimensional structures available [30], which are rigid and devoid of any conformational changes.

Okazaki et al., through the electrophysiological characterization of some cysteine mutants (as Cys2Ala, Cys8Ala, and Cys122Ala), hypothesized the formation of a disulfide bond between the N-terminal Cys2 and the Cys122, located at the bottom of the pore, to explain the decreased VDAC3 channel gating [31]. In a study conducted in our laboratory and reported in Reina et al. [32], a disulfide bond was found between Cys2 and Cys8, either in recombinant human VDAC3 or in the native VDAC3 from rat liver mitochondria. The analysis was performed using a gel-based bottom-up approach and high-resolution mass spectrometry. However, since in this work, the standard procedure, where trypsin digestion is performed at pH 8.3 [23], was used, the results obtained are not unequivocal due to possible disulfide reshuffling under alkaline conditions [33].

More recently, a modification of our gel-free digestion protocol allowed us, for the first time, to characterize intramolecular disulfide bonds in rVDAC2 [34]. In the modified procedure, all sample preparation steps (lysis of mitochondria, enrichment by HTP chromatography, and desalting) were performed under controlled conditions in the pH range 6.5–7.0. Several literature reports demonstrated that under neutral or acidic pH conditions, the formation of non-native disulfide bonds is prevented or at least minimized [33, 35–37]. The successful characterization of disulfide bonds in rVDAC2 prompted us to use the same procedure for a systematic investigation of intramolecular disulfide bonds in rVDAC3 and also to attempt a possible characterization of intermolecular disulfide bonds formed by this protein with other VDAC isoforms.

The ability of VDAC proteins to form dimers, trimers, tetramers, and higher oligomers has been known for some time. The first experimental observations that VDACs can form oligomers have been obtained using NMR [38], double electron–electron resonance spectroscopy (DEER) [12], and atomic force microscopy (AFM) [39, 40].

Dimers have been identified in zebrafish VDAC2 by x-ray crystallography [12] and using a combination of techniques, such as site-directed mutagenesis with cysteine substitutions, transmission electron microscopy, chemical cross-linking, computational analysis, and peptide-based reverse mapping; in all three VDAC regions, highly aggregation-prone dimers were identified [41–43].

Many functional roles of VDACs oligomerization have been predicted, but the exact one(s) are not known. Supramolecular assemblies of VDACs in mitochondrial membranes may provide stability to the protein [11, 41, 42, 44, 45] and may aid in interaction with other proteins such as hexokinase, creatine kinase, and factors that trigger mitochondria-mediated apoptosis [40, 46–48]. A previously unknown function has been attributed to human VDAC1 and VDAC2, involving the import of phospholipids into mitochondria through a scrambling mechanism at the interface of their dimeric beta-barrels [5].

Recently a disulfide bond-mediated dimer of VDAC2 has been designed as the core of an outer membrane structure working as a docking area for PINK1 [6]. Furthermore, little is known about the chemical nature of the interactions responsible for the formation of oligomers in VDAC proteins.

In this work, we have unequivocally demonstrated that disulfide bridges are directly involved in the homo-or hetero-oligomerization of the VDAC isoforms.

## Materials and methods

The chemicals employed during the analysis were of the highest purity commercially available and were used without further purification. Ammonium bicarbonate (AMBIC), TrisHCl, Triton X-100, sucrose, EDTA, HEPES, formic acid (FA), dithiothreitol (DTT), iodoacetamide (IAA), and phenylmethylsulfonyl fluoride (PMSF) were obtained from Aldrich (St. Louis, Missouri, USA), and ammonia was obtained from Carlo Erba (Milan, Italy). Modified porcine trypsin was purchased from Promega (Madison, WI, USA). Water and acetonitrile (OPTIMA LC/MS grade) for LC/MS analyses were provided from Fisher Scientific (Milan, Italy).

### Preparation of VDAC-enriched fractions from rat liver mitochondria

Wistar rats obtained from Charles River Laboratories (Lecco, Italy) were sacrificed. Mitochondria from rat liver were prepared as reported in Saletti et al. (2017) [23]. The experiment was performed in duplicate. All subsequent steps were carried out under carefully controlled pH conditions, in the range between 6.5 and 7.0 (slightly acidic or at most neutral) as reported in Pittalà et al. (2024) [34]. These pH values were chosen to minimize undesirable disulfide reshuffling, which is favored at alkaline pH (7.5–8.5) [49]. To prevent the formation of non-native disulfide bonds, the temperature was also carefully controlled during sample preparation. In our procedure, protein extraction was performed at 4 °C, and samples for disulfide bond analysis by mass spectrometry were prepared at room temperature and then subjected to enzymatic digestion at 37 °C, conditions that avoid cysteine reaction or disulfide bond reshuffling [35].

In detail, 5 mg of intact mitochondria was washed in 10 mM Tris–HCl, 1 mM EDTA at pH 6.5 to eliminate any residue of the extraction buffer [50]. The suspension was then centrifuged for 30 min at 10,000 g at 4 °C, and at the end, after removing the supernatant, the pellet, containing the intact mitochondria, was lysed in buffer A (10 mM Tris–HCl, 1 mM EDTA, 3% Triton at pH 6.5) in a 5:1 ratio (mitochondria mg/buffer volume mL) for 30 min on ice and then centrifuged at 17,400 g for 30 min at 4 °C. The supernatant containing mitochondrial proteins was subsequently loaded onto a homemade glass column 5 × 80 mm, packed with 0.6 g of dry hydroxyapatite (HTP, Bio-Gel, Bio-Rad). The column was eluted with buffer A at 4 °C, and fractions of 500 µL were collected and tested for protein content by a fluorometer assay (Invitrogen QubitTM Protein Assay Kit, Thermo Fisher Scientific, Milan, Italy). Fractions containing proteins were pooled, and the volume was reduced under vacuum to 100 µL. The HTP eluate, enriched in VDAC proteins, was purified from nonprotein contaminating molecules with the PlusOne 2-D Clean-Up Kit (GE Healthcare Life Sciences, Milan, Italy) according to the manufacturer’s instructions.

### In-solution digestion protocol of the HTP eluate from rat liver mitochondria

The desalted protein pellet obtained was suspended in 100 µL of 50 mM ammonium bicarbonate pH 7.0. The protein amount was set to 30 µg using a fluorometer assay (Invitrogen QubitTM Protein Assay Kit, Thermo Fisher Scientific, Milan, Italy). The subsequent steps of free cysteine residues alkylation and enzymatic digestion were performed using a modification of the in-solution sample preparation (reported in Pittalà et al., 2024), originally developed in our laboratory [23, 25]. In detail, the solution containing hydrophobic proteins was directly alkylated in 50 mM ammonium bicarbonate pH 7.0 by the addition of iodoacetamide at a 1:1 molar ratio over the estimated protein thiol groups for 1 h in the dark at 25 °C. Subsequently, digestion was carried out using modified porcine trypsin in 50 mM ammonium bicarbonate pH 7.0 [33] at an enzyme–substrate ratio of 1:50, at 37 °C for 4 h. The digestion was repeated a second time using the same enzyme and the same reaction conditions. The protein digests were then dried under vacuum, dissolved in 25 µL of 5% FA, diluted 1:5 by 5% FA, and analyzed by nanoUHPLC/high-resolution nanoESI-MS/MS.

### Liquid chromatography and tandem mass spectrometry (LC–MS/MS) analysis

In order to assess the reproducibility of the available MS data, mass spectrometry data were acquired in duplicate on an Orbitrap Fusion Tribrid (Q-OT-qIT) mass spectrometer (Thermo Fisher Scientific, Bremen, Germany) equipped with a Thermo Fisher Scientific Dionex UltiMate 3000 RSLCnano system (Sunnyvale, CA). Mass spectrometry data were acquired as described in [34]. In particular, samples obtained by in-solution tryptic digestion were reconstituted in 30 μL of 5% FA aqueous solution, and 1 μL was loaded onto an Acclaim®Nano Trap C18 column (100 μm i.d. × 2 cm, 5 μm particle size, 100 Å). After washing the trapping column with solvent A (H_2_O + 0.1% FA) for 3 min at a flow rate of 7 μL/min, the peptides were eluted from the trapping column onto a PepMap® RSLC C18 EASY Spray, 75 μm × 50 cm, 2 μm, 100 Å column, and were separated by elution at a flow rate of 0.250 μL/min, at 40 °C, with a linear gradient of solvent B (CH_3_CN + 0.1% FA) in A, 5% for 3 min, followed by 5% to 65% in 82 min, followed by 65% to 95% in 5 min, holding 95% B for 5 min, 95% to 5% in 10 min, and re-equilibrating at 5% B for 25 min. Eluted peptides were ionized by a nanospray (Easy-spray ion source, Thermo Scientific) using a spray voltage of 1.7 kV and introduced into the mass spectrometer through a heated ion transfer tube (275 °C). Survey scans of peptide precursors in the m/z range 400–1600 were performed at a resolution of 120,000 (@ 200 m/z) with an AGC target for Orbitrap survey of 4.0 × 10^5^ and a maximum injection time of 50 ms. Tandem MS was performed by isolation at 1.6 Th with the quadrupole, and high energy collisional dissociation (HCD) was performed in the ion routing multipole (IRM), using a normalized collision energy of 35 (a.u.) and rapid scan MS analysis in the ion trap. Only those precursors with charge states 1–5 and an intensity above the threshold of 5·10^3^ were sampled for MS^2^. The dynamic exclusion duration was set to 60 s with a 10 ppm tolerance around the selected precursor and its isotopes. Monoisotopic precursor selection was turned on. AGC target and maximum injection time (ms) for MS/MS spectra were 1.0 × 10^4^ and 100, respectively. The instrument was run in top speed mode with 3 s cycles, meaning the instrument would continuously perform MS^2^ events until the list of non-excluded precursors diminishes to zero or 3 s, whichever is shorter. MS/MS spectral quality was enhanced enabling the parallelizable time option (i.e., by using all parallelizable time during full scan detection for MS/MS precursor injection and detection). Mass spectrometer calibration was performed using the Pierce® LTQ Velos ESI Positive Ion Calibration Solution (Thermo Fisher Scientific). MS data acquisition was performed using the Xcalibur v. 3.0.63 software (Thermo Fisher Scientific).

### Database search analysis

LC–MS/MS data were processed by pLink 2 software [51], PEAKS X-pro de novo sequencing software (Bioinformatics Solutions Inc., Waterloo, ON, Canada), and MaxQuant (MQ) software 2.5.2.0 (https://www.maxquant.org/). The raw data were analyzed and searched against a database containing the three VDAC isoforms of *Rattus norvegicus* (SwissProt Accessions: VDAC1, Q9Z2L0; VDAC2, P81155; VDAC3, Q9R1Z0).

In the pLink 2 software and in the PEAKS X-pro software, full tryptic peptides with a maximum of three missed cleavage sites were subjected to bioinformatic search. Cysteine carboxyamidomethylation was set as a fixed modification, whereas the transformation of N-terminal glutamine and N-terminal glutamic acid residue in the pyroglutamic acid form, acetylation of protein N-terminal, oxidation of methionine, S–S (pLink 2 software), or half of a disulfide bridge (PEAKS X-pro software), with a mass shift of − 1.0078 Da, and trioxidation of cysteine were included as variable modifications. The precursor mass tolerance threshold was 10 ppm, and the max fragment mass error was set to 0.6 Da. In the pLink 2 software, all the other settings were kept as defaults. In the PEAKS X-pro software, peptide spectral matches (PSM) were validated using a Target Decoy PSM Validator node based on *q*-values at a false discovery rate (FDR) ≤ 0.1%. PEAKS score thresholds for PSMs were set to achieve FDR values for PSMs, peptide sequences, and proteins identified below the 0.1% value.

In the MaxQuant software, database search was carried out using the following parameters: (i) tryptic peptides with a maximum of three missed cleavage sites; (ii) cysteine carboxyamidomethylation as a fixed modification; and (iii) acetylation of protein N-terminal, oxidation of methionine, trioxidation and Cys-Cys (mass shift: − 1.0078 Da) of cysteine, and the transformation of N-terminal glutamine and N-terminal glutamic acid residue to pyroglutamic acid form as variable modifications. The decoy mode was “revert.” PSM, protein, and site decoy fraction FDR were set at 0.1% as the threshold for peptide and protein identifications. The minimum score for modified and unmodified peptides was set at 40. All the other parameters were set as default. In the data analysis, only peptides with intensity over the MaxQuant threshold were considered.

The mass spectrometry data have been deposited to ProteomeXchange Consortium (http://proteomecentral.proteomexchange.org) via the PRIDE [52] partner repository with the dataset identifier PXD064110.

To detect and localize disulfide bridges, monoisotopic m/z of possible peptides containing or linked by disulfide bridges were calculated using GPMAW 9.5 software (Lighthouse Data, DK). Then a preliminary search with pLink 2 software [51] was performed in order to identify loop-linked, inter- and intra-protein linked peptides. The MS/MS spectra were visualized by pLabel [51], and then, the spectra of interest were fully annotated by manually searching the full scan spectrum (in high resolution) and the corresponding MS/MS spectrum (in low resolution) by analyzing the raw data with Xcalibur v.3.0.63 software. In addition, Peaks X-pro and MaxQuant software were used to confirm the presence of disulfide bridges linking cysteine residues located in the same tryptic peptide thanks to the variable modifications “half of a disulfide bridge” and “Cys-Cys,” respectively. Both of these PTMs indicate the presence of cysteine residues in the oxidized form to Cys-S˙ (monoisotopic mass: 102.0014 Da). Finally, the MaxLynx algorithm of MaxQuant [53] has been used to detect and localize disulfide-linked intermolecular peptides by searching for disulfide bridge as a cross-linker.

## Results and discussion

**Fig. 1:**
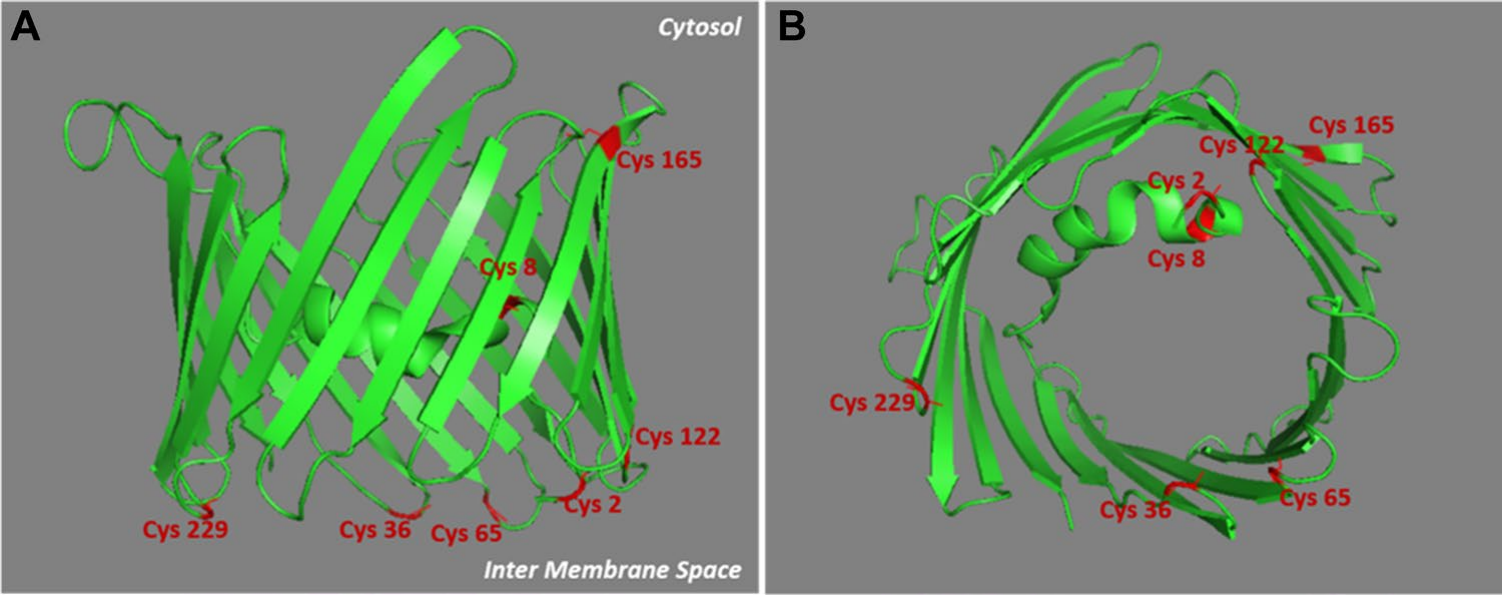
Lateral and top view of rVDAC3. The structure of rVDAC3 is predicted by homology modelling, using mVDAC1 structure (pdb: 3EMN) as a template. Graphical representation was obtained by using PyMOL 1.1 (DeLano Scientific LLC.). Cysteine residues are shown in red

The sequence of rVDAC3 includes seven cysteines in positions 2, 8, 36, 65, 122, 165, and 229 (Fig. 1). The numbering adopted starts from Met1, which, although it is reported in the SwissProt database (Acc. N. Q9R1Z0), is absent in the mature protein [23]. Following post-translational modification, which eliminates the starting methionine, the cysteine encoded as the second amino acid becomes the N-terminal residue. The presence of a cysteine at the beginning of the N-terminal alpha helix is a unique feature of the VDAC3 isoform.

In the homology model built using the mouse VDAC1 as template (Fig. 1), Cys2 points to the intermembrane space (IMS), whereas Cys8, which is in the same N-terminal tryptic peptide with Cys2, is located within the pore. Four of the seven cysteine residues (Cys36, Cys65, Cys122, and Cys229) are located in the loops connecting the β-strands and are therefore protruding towards IMS, as described for Cys2 (Fig. 1). Finally, Cys165 is the only cysteine exposed to the cytosol (Fig. 1).

In the modified procedure adopted for the characterization of the disulfide bonds, following the sample preparation steps (mitochondria lysis, HTP chromatography, and desalting) carried out at a pH of 6.5 to 7.0, the HTP fraction enriched in VDAC proteins was directly alkylated using IAA. The digestion step was performed using trypsin at pH of 6.5 to 7.0, conditions which are known to prevent or at least minimize disulfide scrambling [33, 35–37].The digestion was repeated once considering that a reduction of trypsin activity can be expected under neutral or slightly acidic conditions [33, 35–37]. This procedure allowed obtaining the expected tryptic peptides. Some peptide fragments originating from non-specific cleavages due to a slight increase of the chymotryptic activity of the enzyme in the adopted pH range were also detected [54]. Using the modified experimental conditions, complete sequence coverage of rVCDAC3 was obtained (Figure S1A), identifying the tryptic peptide Arg120-Lys128 and the non-specific peptides Leu114-Asp132 and Val129-Trp141, which contain the previously undetectable Lys115-Phe123 and Gly140-Trp141 stretches [23]. Similarly to what was done in the case of rVDAC2 [34], monoisotopic m/z of potential peptides containing or linked by disulfide bridges in rVDAC3 were calculated using GPMAW 9.5 software. Subsequently, pLink2 and pLabel software were used to perform a preliminary search [51] to find possible peptides linked by intramolecular disulfide bonds and to visualize the MS/MS spectra [51], respectively. The raw data were then analyzed by Xcalibur v.3.0.63 software and processed by PEAKS X-pro and MaxQuant software. Furthermore, the MaxLynx algorithm of MaxQuant [53] was used to detect and localize peptides linked by intermolecular disulfide bonds by searching for the disulfide bridge as a cross-linker. Finally, the spectra of interest were fully annotated by manually searching the full-scan mass spectrum (in high resolution) and the corresponding MS/MS spectrum (in low resolution).

Following this methodology, three intramolecular and seven intermolecular disulfide bonds between rVDAC3 with rVDAC1 and rVDAC2 isoforms were uniquely characterized, as shown in Tables 1 and 4. Furthermore, evidence was obtained for the existence of two additional intramolecular disulfide bonds between Cys2/Cys8 with Cys36 and Cys122, although these identifications were not supported by MS/MS spectra (Table 3). A map indicating the regions of the protein covered by disulfide-linked peptides is shown in Figure S1B.

The identification of the peptides in Tables 1, 3, and 4 is unequivocally supported by the high-resolution measurements, which agreed within 2 ppm with the theoretical values calculated for the disulfide-bonded peptides, and by the MS/MS spectra, in which an almost complete series of y- and b-fragments and other disulfide marker fragments for the proposed structures were observed.

It should be noted that although the three VDACs, present together in the analyzed protein mixture, share large stretches of sequence, the peptides linked by disulfide bonds are unique to each isoform as clearly visible in the Clustal Omega sequence alignment (https://www.ebi.ac.uk/jdispatcher/msa/clustalo) (Figure S2).

To confirm the results obtained and the reproducibility of the experimental data produced, the MS analysis was repeated on a second sample (biological replicate) purified from another set of rat liver mitochondria and subjected to the same procedure (data not shown).

### Intramolecular disulfide bonds

**Fig. 2:**
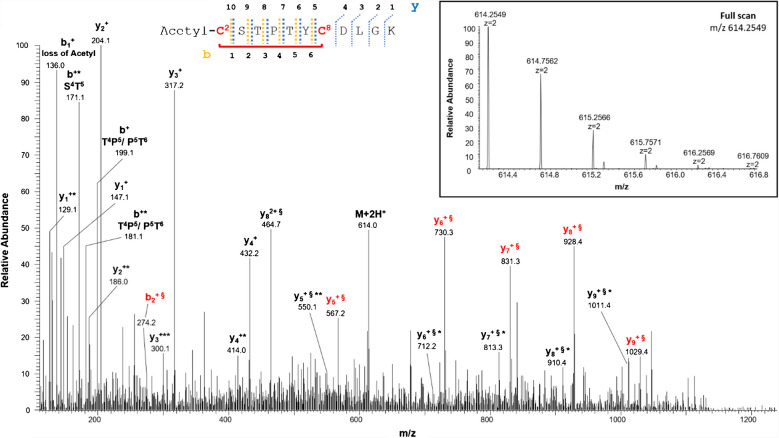
MS/MS spectrum of the doubly charged molecular ion at m/z 614.2549 (calculated 614.2547) of the tryptic peptide 1 (Table 1) of rVDAC3 with the cysteine residues 2 − 8 linked by disulfide bridges. In the peptide sequence, the internal fragments between the disulfide-linked cysteines are shown with yellow dashed lines (b-series) and blue dashed lines (y-series). Fragment ions originated from the neutral loss of H_2_O are indicated by an asterisk. Fragment ions originated from the neutral loss of NH_3_ are indicated by two asterisks. Disulfide-bond-specific fragment ions (cleaved at sulfur-carbon bonds) are indicated by § and in red. The inset shows the full scan mass spectrum of the molecular ion

HRMS analysis of the sample led to the identification of a disulfide bridge in the N-terminal domain between the cysteines 2, in the acetylated form, and 8 (Table 1, peptide 1). The structure of the peptide is supported by the full-scan mass spectrum and the corresponding MS/MS spectrum of the doubly charged molecular ion at m/z 614.2549 of the N-terminal tryptic peptide (Table 1) containing the disulfide bond C2-8 reported in Fig. 2. A doubly charged molecular ion due to the same fragment with both cysteines in the carboxyamidomethylated form and Cys2 acetylated was also detected at m/z 672.2845 (Table S1, peptide 1, and Figure S3).

**Table 1:**
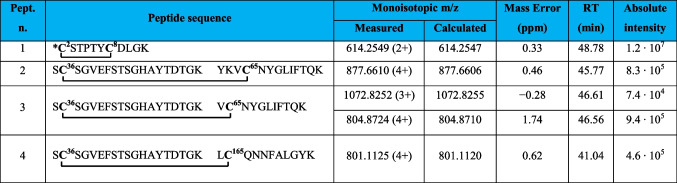
Intramolecular disulfide bridged peptides in VDAC3 from *Rattus norvegicus*

*N-terminal cysteine acetylated; **C**, cysteine in the oxidized form of disulfide bridge

A comparison of the absolute intensities of the doubly charged molecular ion of disulfide-bonded peptide 1 (Table 1) and that of the doubly charged molecular ion of peptide 2 (Table 2) provides a rough estimate of the relative abundance of the two cysteine forms and indicates that the disulfide-bonded fragment 1 is predominant over the reduced one, which appears to be present only in trace amounts (Table 2).

**Table 2:**
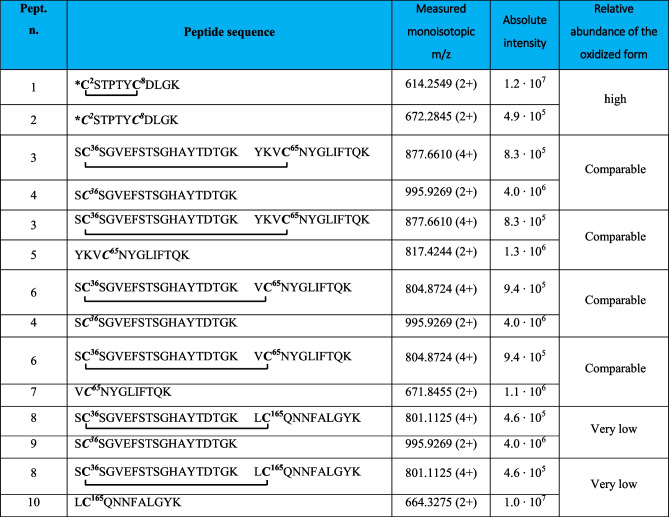
Comparison of relative molecular ions intensities of peptides containing disulfide bridged cysteines and corresponding peptides containing carboxyamidomethylated cysteines

***C***, cysteine carboxyamidomethylated; **C**, cysteine in the oxidized form of disulfide bridge. Further information on the carboxyamidomethylated peptides of rVDAC3 is given in Table S1

**Fig. 3:**
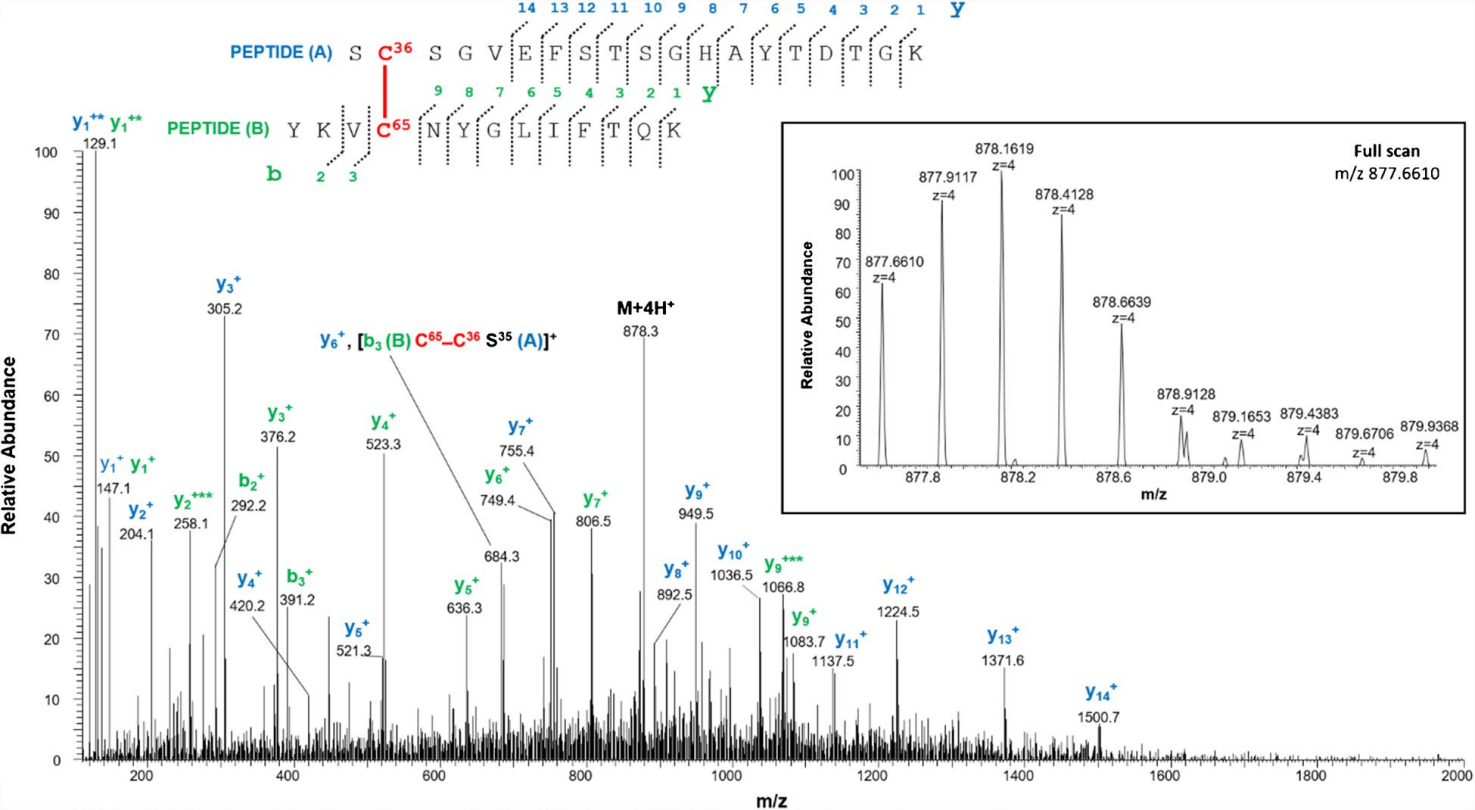
MS/MS spectrum of the quadruply charged molecular ion at m/z 877.6610 (calculated 877.6606) of tryptic peptide 3 (Table 1) of rVDAC3 with cysteine residues 36 and 65 linked by a disulfide bridge. The fragments with intact disulfide bond are reported in the MS/MS spectrum. Fragments from peptide (**A**) and peptide (**B**) are indicated in blue and green, respectively. Fragment ions originated from the neutral loss of H_2_O are indicated by an asterisk. Fragment ions originated from the neutral loss of NH_3_ are indicated by two asterisks. The inset shows the full scan mass spectrum of the molecular ion

A disulfide bond involving Cys36 and Cys65 is demonstrated by the quadruply charged molecular ion of peptide 2 (Table 1) at m/z 877.6610, where the Tyr62-Lys74 fragment contains a missed cleavage. The full scan mass spectrum and the corresponding MS/MS spectrum are shown in Fig. 3. Furthermore, this identification is also confirmed by the detection of the triply and quadruply charged molecular ions of peptide 3 (Table 1) at m/z 1072.8252 (Figure S4A) and 804.8724 (Figure S4B), respectively, and by their MS/MS spectra.

The cross-linked and non-cross-linked peptides are present in comparable amounts as indicated by the relative intensities of the quadruply charged molecular ions of the fragments 3 and 6 (Table 2) compared to those of the doubly charged molecular ions of fragments 4, 5, and 7 (Table 2); although it should be noted that this assessment is very approximate because, due to the very different structure and mass of the two peptides, they certainly have different ionization efficiency values.

**Fig. 4:**
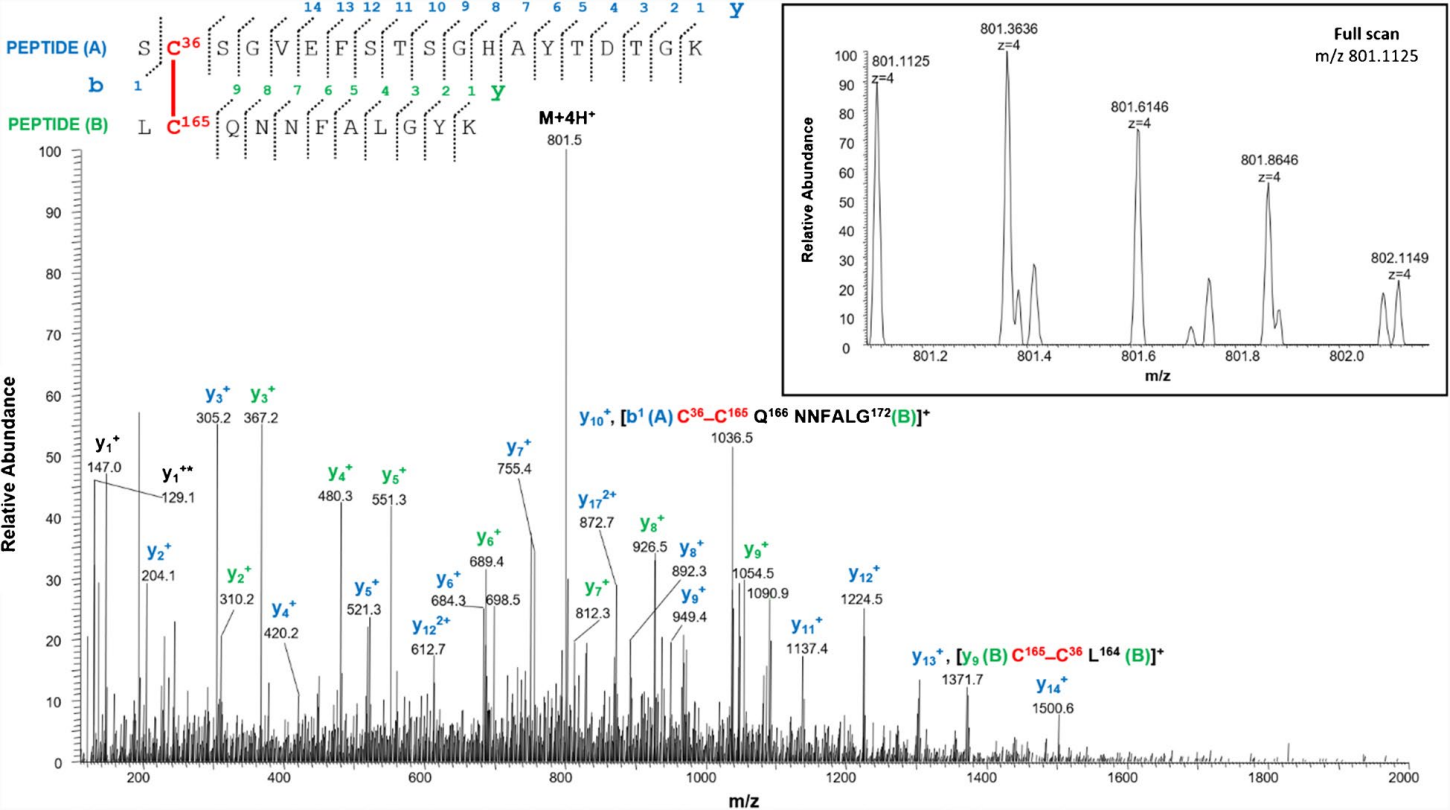
MS/MS spectrum of the quadruply charged molecular ion at m/z 801.1125 (calculated 801.1120) of tryptic peptide 4 (Table 1) of rVDAC3 with cysteine residues 36 and 165 linked by a disulfide bridge. The fragments with intact disulfide-bond are reported in the MS/MS spectrum. Fragments from peptide (**A**) and peptide (**B**) are indicated in blue and green, respectively. Fragment ions originated from the neutral loss of H_2_O are indicated by an asterisk. Fragment ions originated from the neutral loss of NH_3_ are indicated by two asterisks. The inset shows the full scan mass spectrum of the molecular ion

In the predicted three-dimensional conformation of rVDAC3 (Fig. 1), Cys36 and Cys65, located in the loops exposed to oxidative IMS, are in a suitable position to form a disulfide bridge. A different case occurs for Cys36 and Cys165. Indeed, based on the predicted structural model built by homology from mouse VDAC1 (Fig. 1), Cys165 is the only cysteine that faces the cytosol and therefore could not be able to bind via a disulfide linkage with Cys36 residue located in the IMS, because the two residues are extremely distant. Despite this observation, our results show that Cys36 and Cys165 are joined by a disulfide bond, as unequivocally demonstrated by the detection of the quadruply charged molecular ion at m/z 801.1125 (Table 1, peptide 4, and Fig. 4).

Consequently, it should be assumed that the reconstructed three-dimensional structure of rVDAC3 (Fig. 1) might be incorrect or, more likely, the Cys36-Cys165 bond is an intermolecular disulfide bond formed between two different rVDAC3 molecules, whose spatial arrangement, however, cannot be derived from the present data.

The carboxyamidomethylated form of Cys165 was also identified (Table 2, peptide 10), and comparison of the absolute intensities of the corresponding molecular ions (Table 2, peptides 8–10) indicates that the disulfide bridge form (Table 2, peptides 8 and 9) is present in much lower amounts than the reduced one.

Evidence for the formation of two additional intramolecular disulfide bonds was provided by the detection of the molecular ions of the corresponding peptides, the exact masses of which were in excellent agreement with the calculated values (Table 3).

**Table 3:**
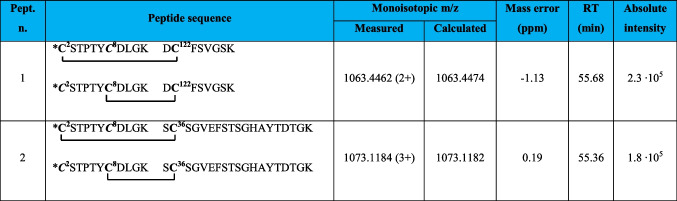
Intramolecular disulfide bridged peptides in VDAC3 from Rattus norvegicus not supported by MS/MS

*N-terminal cysteine acetylated. **C**, cysteine carboxyamidomethylated; **C**, cysteine in the oxidized form of disulfide bridge

Evidence for the formation of a disulfide bond between Cys2 or Cys8 and Cys122 is provided by the detection of the doubly charged molecular ion of peptide 1 (Table 3), at m/z 1063.4462. In this peptide, one of the two cysteines present in the N-terminal portion of rVDAC3 forms a disulfide bond with Cys122, while the other cysteine is carboxyamidomethylated, indicating that it is present in a reduced form in the native protein. No MS/MS was obtained corresponding to this molecular ion, probably due to its low absolute intensity, and thus, it was not possible to identify which of the two N-terminal cysteines is bound to Cys122. Likewise, the detection of a triply charged molecular ion at m/z 1073.1184 corresponding to peptide 2, Table 3, indicates the formation of a disulfide bond between Cys2 or Cys8 and Cys36. Also, for this molecular ion, no MS/MS spectrum was acquired, most likely for the same reasons seen above. The low absolute intensities of these two molecular ions could be due to the presence of a very low amount of the corresponding peptides in the analyzed mixture. However, when evaluating these results, one should take into account that if one of the two N-terminal cysteines is linked to Cys122 or Cys36, the second one could be in the reduced state in the native protein, thus being carboxyamidomethylated, as occurs in the detected peptides, but it could also be in another oxidation state, such as trioxidized to sulfonic acid or involved in another disulfide bond with another cysteine-containing peptide. While the possibility that the second cysteine is trioxidized can be excluded since the molecular ions corresponding to peptides 1 and 2 (Table 3) with a trioxidized cysteine were not present, the formation of a disulfide bond with an unknown cysteine-containing peptide cannot be excluded.

### Intermolecular disulfide bonds

Mass spectral data confirmed the existence of supramolecular structures of VDACs due to the formation of intermolecular disulfide bonds. In this paper, we focused on the analysis of oligomeric structures between rVDAC3 and the other two isoforms (see Figures S5 and S6 for rVDAC1 and rVDAC2 predicted structures, respectively). This investigation led to the characterization of five disulfide bonds between rVDAC3 and rVDAC1, and two S–S bonds between rVDAC3 and rVDAC2 (Table 4).

**Fig. 5:**
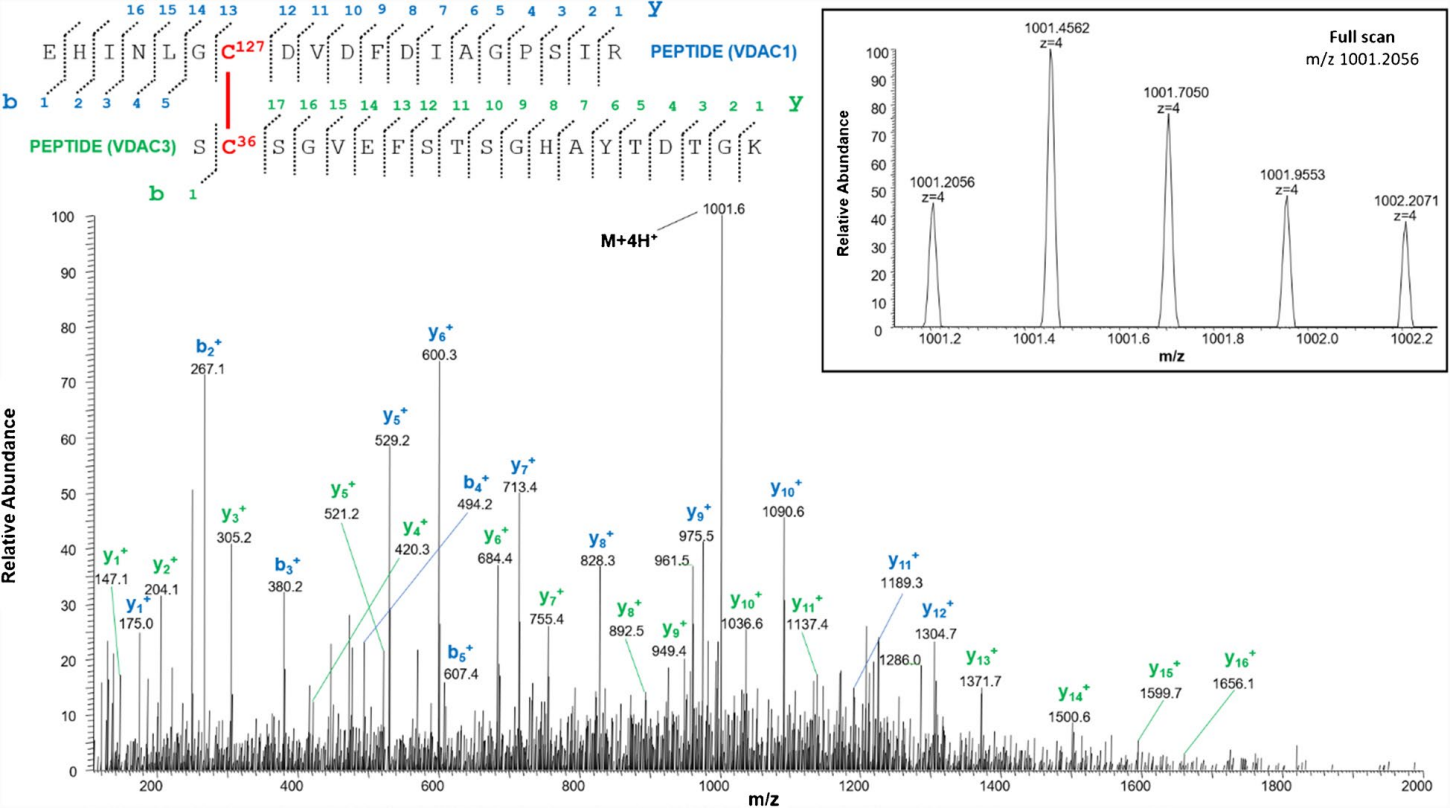
MS/MS spectrum of the quadruply charged molecular ion at m/z 1001.2056 (calculated 1001.2036) of tryptic peptide 1 (Table 3) with cysteine residues 127 of rVDAC1 and 36 of rVDAC3 linked by a disulfide bond. Fragments from peptide (VDAC1) and peptide (VDAC3) are indicated in blue and green, respectively. Fragment ions originated from the neutral loss of H_2_O are indicated by an asterisk. Fragment ions originated from the neutral loss of NH_3_ are indicated by two asterisks. The inset shows the full scan mass spectrum of the molecular ion

In detail, the presence of the quadruply charged molecular ion at m/z 1001.2056 (Table 4, peptide 1, and Fig. 5) allowed us to demonstrate that Cys36 contained in the tryptic peptide S^35^CSGVEFSTSGHAYTDTGK^53^ of rVDAC3 is linked to Cys127 of the tryptic peptide E^121^HINLGCDVDFDIAGPSIR^139^ of rVDAC1.

**Table 4:**
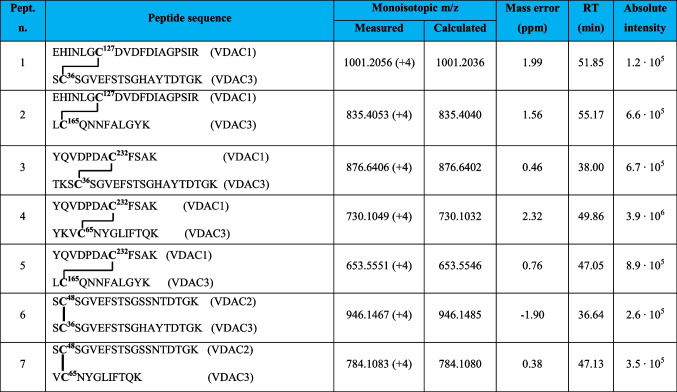
Intermolecular disulfide bridged in VDACs from *Rattus norvegicus*

**C**, cysteine in the oxidized form of disulfide bridge

The same cysteine residue 127 of the rVDAC1 is also engaged in a disulfide bond with Cys165 of rVDAC3 as documented by the quadruply charged molecular ion at m/z 835.4053 (Table 4, peptide 2, and Figure S7).

Furthermore, Cys232 of rVDAC1, included in the tryptic peptide Y^225^QVDPDACFSAK^236^, forms disulfide bonds with cysteines 36, 65, and 165 of rVDAC3.

Indeed, the disulfide bond between Cys232 and Cys36 (peptide sequence T^33^KSCSGVEFSTSGHAYTDTGK^53^) is demonstrated by the detection of the quadruply charged molecular ion at m/z 876.6406 (Table 4, peptide 3); the quadruply charged molecular ion at m/z 730.1049 (Table 4, peptide 4) documents the formation of the disulfide bond between Cys232 and Cys65 (peptide sequence Y^62^KVCNYGLIFTQK^74^); finally, the quadruply charged molecular ion at m/z 653.5551 indicates the formation of a disulfide bridge with Cys165 (peptide sequence L^164^CQNNFALGYK^174^) (Table 4, peptide 5). The corresponding full scan and MS/MS spectra are shown in Figures S8, S9, and S10, respectively.

The two disulfide bonds between cysteines 36 (peptide sequence S^35^CSGVEFSTSGHAYTDTGK^53^) and 65 (peptide sequence V^64^CNYGLIFTQK^74^) of rVDAC3 with Cys48 in the tryptic segment S^47^CSGVEFSTSGSSNTDTGK^65^of rVDAC2 were supported by the detection of the respective quadruply charged molecular ions at m/z 946.1467 (Table 4, peptide 6, and Figure S11) and 784.1083 (Table 4, peptide 7, and Figure S12).

In order to have a rough estimate of the relative abundance of the cross-linked and non-cross-linked fragments, a comparison of their relative intensities was performed (Table 5). The results indicate that the disulfide cross-linked peptide comprising Cys127 of rVDAC1 and Cys36 of rVDAC3 (Table 5, peptide 1) is present in a very low amount. Also, cross-linked fragments between Cys48 of rVDAC2 and both cysteines 36 and 65 of rVDAC3 (Table 5, peptides 12 and 14) are detected in a very low amount. In contrast, the disulfide cross-linked peptide between Cys232 of rVDAC1 and Cys65 of rVDAC3 and the corresponding reduced peptides are detected in comparable amounts (Table 4, peptide 9). The other identified cross-linked fragments involving peptides of both rVDAC1 and rVDAC3 are found in low amounts (Table 5, peptides 4, 6, and 11).

**Table 5:**
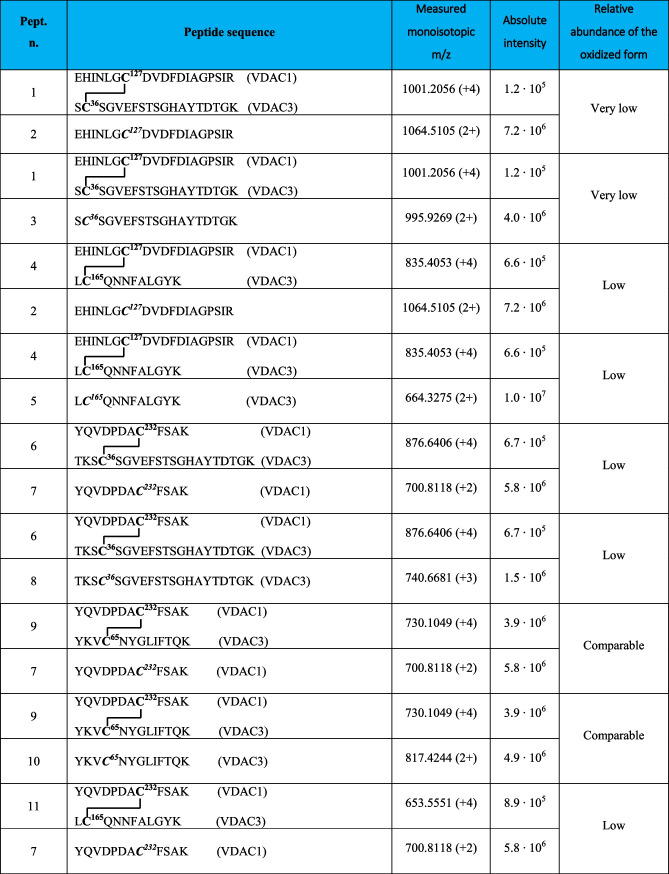

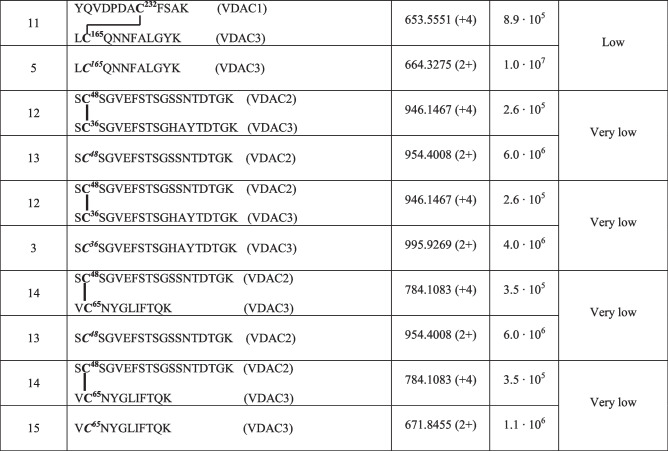
Comparison of relative molecular ions intensities of peptides containing disulfide-bridged cysteines and corresponding peptides containing carboxyamidomethylated cysteines

**C**, cysteine carboxyamidomethylated; **C**, cysteine in the oxidized form of disulfide bridge. Further information on the carboxyamidomethylated peptides of rVDAC1 and rVDAC2 is given in Tables S2 and S3

### Comparison of redox pattern of cysteines in rVDAC2 and rVDAC3

**Fig. 6:**
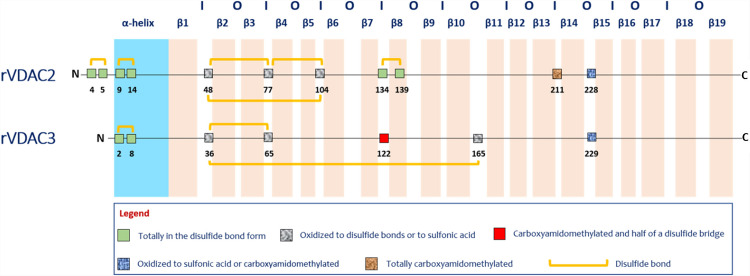
Comparison of redox pattern of VDAC2 and VDAC3 from *Rattus norvegicus*. The N-terminal α-helix and the β-strands are shown in blue and pink, respectively. The internal loops, exposed to the intermembrane space, are indicated with I. The outer loops, exposed to the cytosol, are indicated with O

The oxidative pattern of cysteines in rVDAC3 obtained in the present investigation was compared with that previously obtained for rVDAC2. The comparison is summarized in Fig. 6. It is very interesting to note that, in the two sequences, the homologous cysteine residues are in the same oxidation state. In particular, cysteines 2 and 8 of rVDAC3, like cysteines 9 and 14 of rVDAC2 [34], which are all located in the N-terminal α-helix region, are linked by disulfide bonds (Figs. 1 and S6). This finding suggests that at least one disulfide bridge at the N-terminus of VDAC2 and VDAC3 isoforms is required because it may have a specific function. Given its location, this disulfide bond may be indispensable to keep the N-terminal α-helix close to the channel opening in the IMS in order to regulate ions and metabolites exchange [32].

In rVDAC3, Cys2 and Cys8 were also identified in the carboxyamidomethylated form (Figure S3, Table S1, peptide 1), even if comparison of the absolute molecular ion intensities of the oxidized disulfide-bridged versus the reduced one indicates that the latter is present in a very small amount (Table 2).

Similarly, cysteines 36, 65, and 165 of rVDAC3 as well as the homologous cysteines 48, 77, and 104 of rVDAC2, which are located in the loops connecting the β-strands (Figs. 1 and S6), are oxidized to both sulfonic acid (Table S4, peptides 1–6, and Figures S13-S15) and disulfide bridges (Table 1, Figs. 3, 4, and S4). Furthermore, the cysteines 36, 65, and 165 (Table S1, peptides 2, 3, 4, 6, and 7, and Figures S16-S18) are also detected in the reduced form in considerable amounts (Tables 2 and S5).

Cys122 of rVDAC3, located in a loop region exposed to IMS (Fig. 1), is homologous to Cys134 of rVDAC2, which is linked to Cys139 by a disulfide bond particularly resistant to reduction [34]. A cysteine residue homologous to Cys139 of rVDAC2 does not exist in rVDAC3. As reported in Fig. 6, in this study Cys122 is detected as carboxyamidomethylated (Table S1, peptide 5, Figure S19), indicating that it is present partially in the reduced form in the native protein, but also with the variable “Half of a disulfide bridge” modification (Table S6, Figure S20), suggesting that it is engaged in a disulfide bond, although the cysteine residue to which it is bound is unknown. The predictable peptides corresponding to the formation of a disulfide bond with another rVDAC3 molecule or another rVDAC isoform were not found; therefore, the disulfide bond in which Cys122 is involved cannot be defined [55].

Finally, Cys229 of rVDAC3 and its homologous Cys228 of rVDAC2, which, based on the predicted three-dimensional structure, are located in the IMS-exposed region (Figs. 1 and S6), are always predominantly oxidized to sulfonic acid [23] (Table S4, peptide 7, and Figure S21), while the corresponding reduced forms are present only in trace amounts (Table S1, peptide 8, Table S5, and Figure S22).

The formation of intermolecular disulfide bonds helps to explain the previously observed VDAC oligomerization [56]. Oligomerization of more VDAC monomers has been proposed to allow the formation of a supramolecular larger pore, which can be used as a conduit large enough to allow the exit of DNA mt molecules or fragments [57], and to contribute to the permeability transition [58]. Multimeric pores of sufficiently large diameters could release cytochrome *c* from the mitochondrial IMS to the cytosol, thus triggering mitochondria-mediated apoptosis [56]. Forces and bonds involved in the VDAC oligomerization expect precise identification. Intermolecular disulfide bridges could contribute to the formation or stabilization of oligomers. A suggestion in this sense has been recently proposed in [6].

## Conclusions

A modification of a gel-free digestion protocol, in which all sample preparation steps (lysis of mitochondria, enrichment by HTP chromatography, and desalting) were performed under controlled conditions in the pH range of 6.5 to 7.0 to prevent the formation of non-native disulfide bonds, coupled with nUHPLC/HR nESI-MS/MS analysis, was used for a systematic investigation of intramolecular disulfide bonds in rVDAC3 and also to attempt a possible characterization of intermolecular disulfide bonds formed by this protein with other VDAC isoforms. As a result, three intramolecular and seven intermolecular disulfide bonds between rVDAC3 and rVDAC1 and rVDAC2 isoforms were uniquely characterized. Furthermore, evidence was obtained for the existence of two additional intramolecular disulfide bonds between Cys2/Cys8 and Cys36 and Cys122, although these identifications were not supported by MS/MS spectra. In evaluating these results, it is worth emphasizing that the experimental procedure used in this work is the one currently adopted for the direct characterization of disulfide bonds, even if the possibility of the presence of non-native disulfide bonds, as a result of non-native conformations during sample preparation and tryptic digestion, cannot be completely ruled out. It must also be considered that the sample preparation steps adopted (mitochondria lysis, HTP chromatography, and desalting) are known to maintain the native conformations of VDACs. This preparation procedure is in fact used to obtain samples for subsequent functional studies [59–61].

It is becoming increasingly clear that cysteines in VDACs are important in sensing and responding to oxidative stress and, more generally, in modulating VDACs’ activity. The direct structural characterization of intra- and intermolecular disulfide bonds in rVDAC3, adding precise molecular details, significantly complements the results of previous investigations aimed at elucidating the role of cysteines in VDACs, obtained using methods such as mutagenesis or molecular dynamics simulations. This knowledge certainly clarifies aspects of the molecular structure that were still controversial in recent literature, thus allowing a better understanding of their role in proteins’ activity and paving the way for the development of possible modulation methods. Further investigations appear to be desirable, particularly into the identification of hetero intermolecular disulfide bridges.

## Supplementary Information

Below is the link to the electronic supplementary material.

Supplementary file1 (DOCX 4430 KB)

## Data Availability

Data not included in the paper are available via ProteomeXchange with identifier PXD064110.
