# Supplementary material for: Structural characterization of intra- and intermolecular disulfide bonds in voltage-dependent anion channel 3 (VDAC3) protein from Rattus norvegicus by high-resolution mass spectrometry
Source: Anal Bioanal Chem. 2025 Aug 28;417(24):5555–70. doi: 10.1007/s00216-025-06074-w (PMC12521285; doi:10.1007/s00216-025-06074-w)
Supplement: Supplementary file 1 — Supplementary file1 (DOCX 4430 KB) [file 216_2025_6074_MOESM1_ESM.docx]

**Supporting Information**

**Structural characterization of intra- and intermolecular disulfide bridges in Voltage-Dependent Anion Channel 3 (VDAC3) protein from *Rattus norvegicus* by high resolution mass spectrometry**

Maria Gaetana Giovanna Pittalà^1^, Annamaria Cucina^1^, Stefano Conti-Nibali^2^, Vincenzo Cunsolo^1^, Antonella Di Francesco^1^, Giuseppe Battiato^2^, Simona Reina^2^, Salvatore Foti^1^, Vito De Pinto^2^ and Rosaria Saletti^1,*^

^1^Department of Chemical Sciences, Organic Mass Spectrometry Laboratory, University of Catania, Viale A. Doria 6, 95125 Catania, Italy;

^2^Department of Biomedical Sciences and Biotechnology, Section of Biology and Genetics, University of Catania, via S. Sofia, 97, 95123, Catania, Italy.

**Correspondence to:* Rosaria Saletti; *e-mail:* [rsaletti@unict.it](mailto:rsaletti@unict.it)

**Table S1. Tryptic peptides found in rVDAC3 after carboxyamidomethylation.**

Retention time, experimentally measured and calculated monoisotopic m/z of the molecular ions, positions in the sequence and the peptide sequence of fragments present in the tryptic digest of carboxyamidomethylated rVDAC3 are reported. All sequences were confirmed by MS/MS.

| **Pept.**  **n.** | **RT**  **(min)** | **Monoisotopic m/z** | | **Position in the sequence** | **Peptide sequence** |
| --- | --- | --- | --- | --- | --- |
|  |  | **Measured** | **Calculated** |  |  |
| 1 | 44.25 | 672.2841 (2+) | 672.2839 | 2-12 | ****C^2^***STPTY***C^8^***DLGK |
| 2 | 33.36 | 995.9269 (2+) | 995.9260 | 35-53 | S***C^36^***SGVEFSTSGHAYTDTGK |
| 3 | 47.85 | 817.4244 (2+) | 817.4240 | 62-74 | YKV***C^65^***NYGLIFTQK |
| 4 | 51.81 | 671.8455 (2+) | 671.8448 | 64-74 | V***C^65^***NYGLIFTQK |
| 5 | 31.90 | 528.2524 (2+) | 528.2511 | 120-128 | RD***C^122^***FSVGSK |
| 6 | 37.52 | 771.8912 (2+) | 771.8903 | 162-174 | SKL***C^165^***QNNFALGYK |
| 7 | 44.01 | 664.3275 (2+) | 664.3268 | 164-174 | L***C^165^***QNNFALGYK |
| 8 | 23.60 | 441.7162 (2+) | 441.7167 | 225-230 | YRLD***C^229^***R |

***C**: N-terminal acetylated; ***C***: cysteine carboxyamidomethylated.

**Table S2. Tryptic peptides found in rVDAC1 after carboxyamidomethylation.**

Retention time, experimentally measured and calculated monoisotopic m/z of the molecular ions, positions in the sequence and the peptide sequence of fragments present in the tryptic digest of carboxyamidomethylated rVDAC1 are reported. All sequences were confirmed by MS/MS.

| **Pept.**  **n.** | **RT**  **(min)** | **Monoisotopic m/z** | | **Position in the sequence** | **Peptide sequence** |
| --- | --- | --- | --- | --- | --- |
|  |  | **Measured** | **Calculated** |  |  |
| 1 | 55.39 | 1064.5105 (2+) | 1064.5105 | 121-139 | EHINLG***C^127^***DVDFDIAGPSIR |
| 2 | 41.18 | 700.8118 (+2) | 700.8112 | 225-236 | YQVDPDA***C^232^***FSAK |

***C***: cysteine carboxyamidomethylated.

**Table S3. Tryptic peptide found in rVDAC2 after carboxyamidomethylation.**

Retention time, experimentally measured and calculated monoisotopic m/z of the molecular ion, position in the sequence and the peptide sequence of fragment present in the tryptic digest of carboxyamidomethylated rVDAC2 is reported. The sequence was confirmed by MS/MS.

| **Pept.**  **n.** | **RT**  **(min)** | **Monoisotopic m/z** | | **Position in the sequence** | **Peptide sequence** |
| --- | --- | --- | --- | --- | --- |
|  |  | **Measured** | **Calculated** |  |  |
| 1 | 32.86 | 954.4008 (2+) | 954.3998 | 47-65 | S***C^48^***SGVEFSTSGSSNTDTGK |

***C***: cysteine carboxyamidomethylated.

**Table S4. Sulfur-modified peptides found in rVDAC3 tryptic digest after carboxyamidomethylation.**

Retention time, experimentally measured and calculated monoisotopic m/z of the molecular ions, position in the sequence and peptide sequence of sulfur containing tryptic fragments found in the analysis of rVDAC3 carboxyamidomethylated and digested in-solution.

| **Pept.**  **n.** | **RT**  **(min)** | **Monoisotopic m/z** | | **Position in the sequence** | **Peptide sequence** |
| --- | --- | --- | --- | --- | --- |
|  |  | **Measured** | **Calculated** |  |  |
| 1 | 31.52 | 1105.9794 (2+) | 1105.9792 | 33-53 | TKS**C^36^**SGVEFSTSGHAYTDTGK |
| 2 | 36.95 | 991.4077 (2+) | 991.4079 | 35-53 | S**C^36^**SGVEFSTSGHAYTDTGK |
| 3 | 53.87 | 812.9072 (2+) | 812.9059 | 62-74 | YKV**C^65^**NYGLIFTQK |
| 4 | 58.54 | 667.3283 (2+) | 667.3267 | 64-74 | V**C^65^**NYGLIFTQK |
| 5 | 43.67 | 767.3725 (2+) | 767.3722 | 162-174 | SKL**C^165^**QNNFALGYK |
| 6 | 52.04 | 659.8088 (2+) | 659.8087 | 164-174 | L**C^165^**QNNFALGYK |
| 7 | 26.02 | 437.1976 (2+) | 437.1983 | 225-230 | YRLD**C^229^**R |

**C**: cysteine oxidized to sulfonic acid; ***C***: cysteine carboxyamidomethylated.

**Table S5.** Comparison of the absolute intensities of molecular ions of selected sulfur containing tryptic peptides found in the analysis of rVDAC3, carboxyamidomethylated and digested in-solution.

| **Peptide** | **Position in the sequence** | **Measured monoisotopic *m/z*** | **Absolute intensity** | **Relative abundance of the oxidized form** |
| --- | --- | --- | --- | --- |
| S**C^36^**SGVEFSTSGHAYTDTGK | 35-53 | 991.4077 (2+) | 7.4 ∙ 10^6^ | 1.9 |
| S***C^36^***SGVEFSTSGHAYTDTGK |  | 995.9269 (2+) | 4.0 ∙ 10^6^ |  |
| YKV**C^65^**NYGLIFTQK | 62-74 | 812.9072 (2+) | 3.9 ∙ 10^6^ | 1 |
| YKV***C^65^***NYGLIFTQK |  | 817.4244 (2+) | 3.9 ∙ 10^6^ |  |
| V**C^65^**NYGLIFTQK | 64-74 | 667.3283 (2+) | 3.1 ∙ 10^5^ | 0.3 |
| V***C^65^***NYGLIFTQK |  | 671.8455 (2+) | 1.1 ∙ 10^6^ |  |
| L**C^165^**QNNFALGYK | 164-174 | 659.8088 (2+) | 1.1 ∙ 10^6^ | 0.1 |
| L***C^165^***QNNFALGYK |  | 664.3275 (2+) | 1.0 ∙ 10^7^ |  |
| YRLD**C^229^**R | 225-230 | 437.1976 (2+) | 4.6 ∙ 10^5^ | 4.6 |
| YRLD***C^229^***R |  | 441.7162 (2+) | 1.0 ∙ 10^5^ |  |

**C**: cysteine oxidized to sulfonic acid; ***C***: cysteine carboxyamidomethylated.

**Table S6. Sulfur-modified peptide found in rVDAC3 tryptic digest after carboxyamidomethylation.**

Retention time, experimentally measured and calculated monoisotopic m/z of the molecular ion, position in the sequence and the peptide sequence of fragment present in the tryptic digest of carboxyamidomethylated rVDAC3 is reported. The sequence was confirmed by MS/MS.

| **Pept.**  **n.** | **RT**  **(min)** | **Monoisotopic m/z** | | **Position in the sequence** | **Peptide sequence** |
| --- | --- | --- | --- | --- | --- |
|  |  | **Measured** | **Calculated** |  |  |
| 1 | 39.18 | 720.0279 (3+) | 720.0332 | 114-132 | LKASYRRD**C^122^**FSVGSKVDID |

**C**: Half of a disulfide bridge.


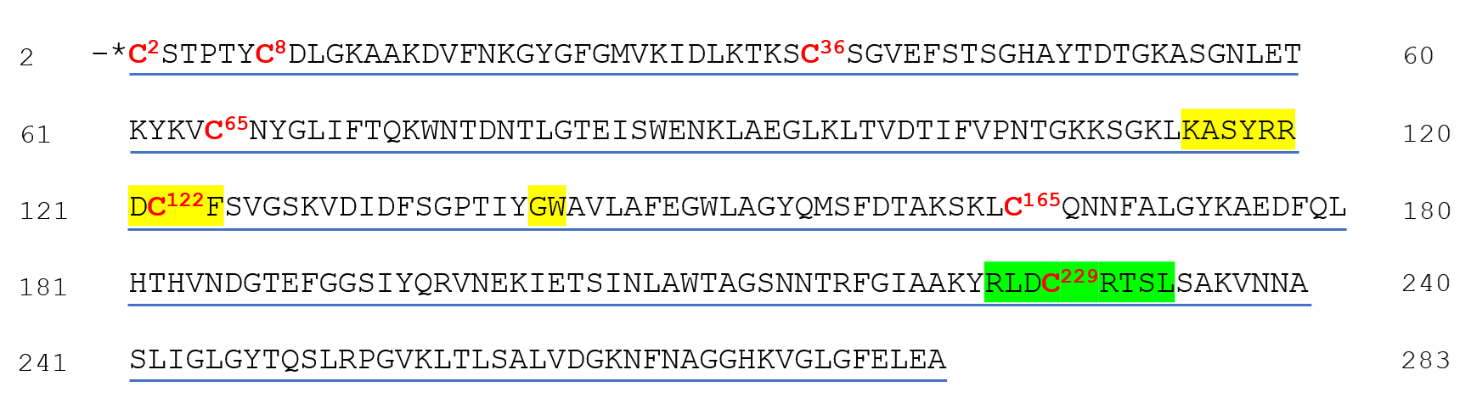


**Figure S1A..** Sequence coverage map of rVDAC3 obtained by the experimental procedure reported in the text (see Materials and Methods). Cysteine residues are shown in red. Covered sequences are underlined in blue. The sequence fragments not covered in our previously work [23] are highlighted in yellow. Instead, the fragment shown in green and containing the cysteine residue 229 had been found only as sulfonic acid exclusively in the chymotryptic digest [23].

* acetylation; - absence of the N-terminal methionine.


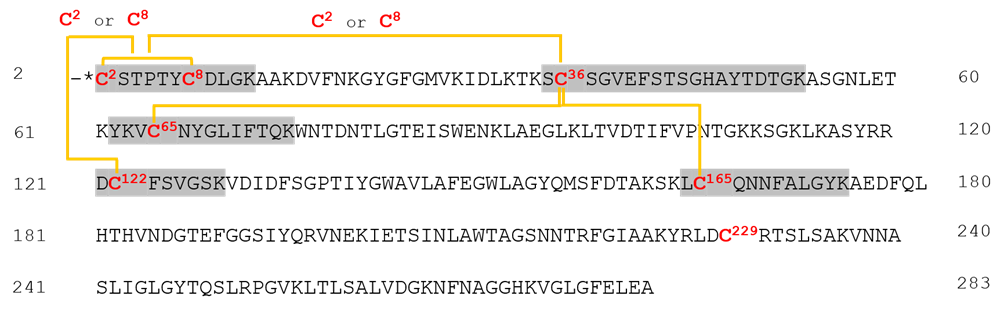


**Figure S1B.** Map of disulfide bonds in the VDAC3 protein. Cysteine residues are shown in red; peptides involved in disulfide bonds are highlighted in grey; disulfide bonds are indicated by yellow lines.

* acetylation; - absence of the N-terminal methionine.


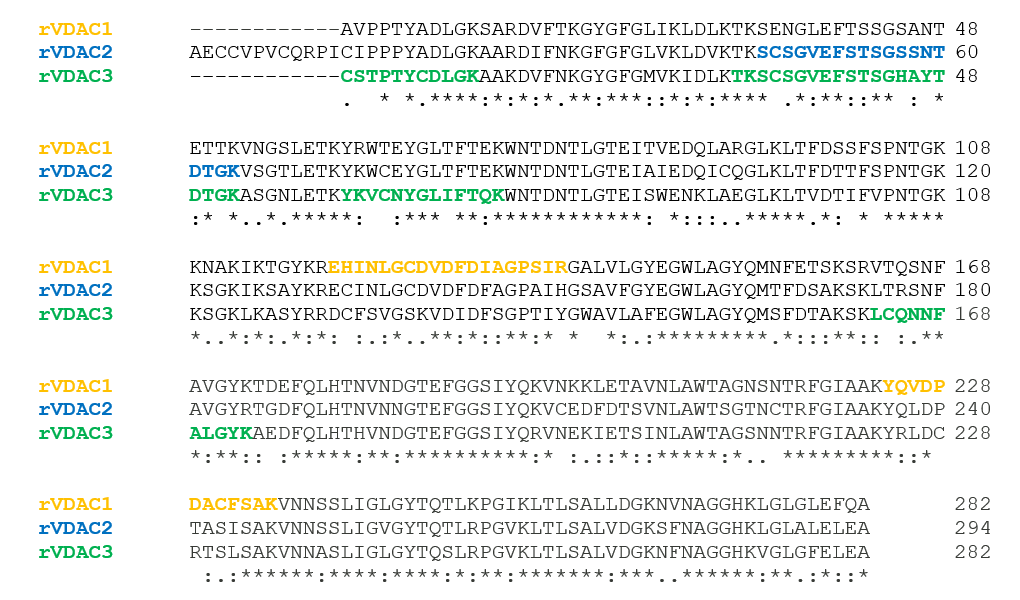


**Figure S2.** Alignments of rat VDAC isoforms by Clustal Omega (https://www.ebi.ac.uk/jdispatcher/msa/clustalo). Disulfide bridged peptides are indicated in yellow, blue and green for rVDAC1, rVDAC2 and rVDAC3, respectively. Conserved amino acids are marked by an asterisk; non identical amino acids are not marked or marked by one or two dots, depending on the structure analogy.


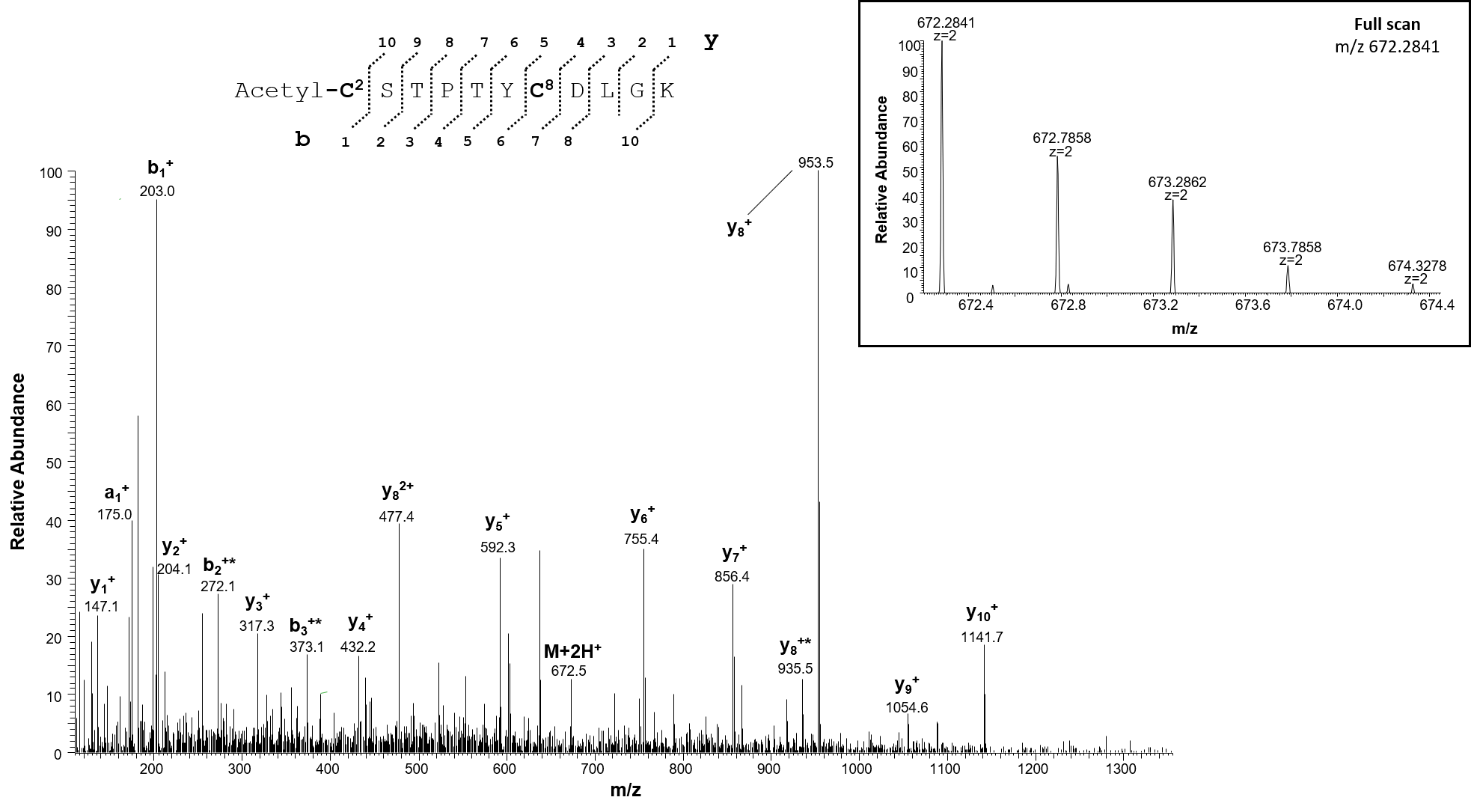


**Figure S3.** MS/MS spectrum of the doubly charged molecular ion at m/z 672.2841 (calculated 672.2839) of the tryptic peptide 2 (Table 2) of rVDAC3 with the cysteine residues 2−8 in the carboxyamidomethylated form and with Cys2 in the acetylated form. Fragment ions originated from the neutral loss of H_2_O are indicated by an asterisk. Fragment ions originated from the neutral loss of NH_3_ are indicated by two asterisks.

**A)**


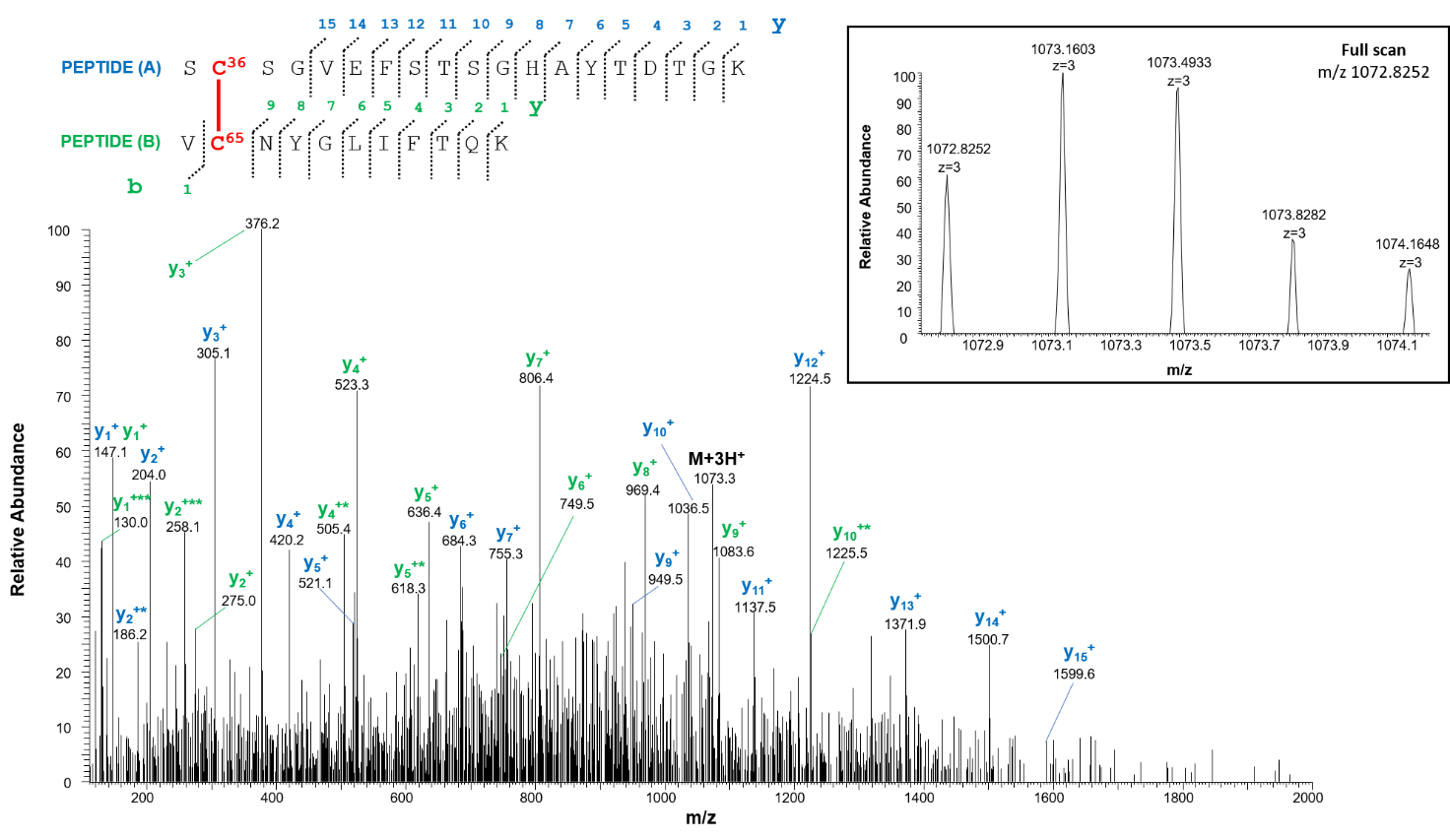


**B)**


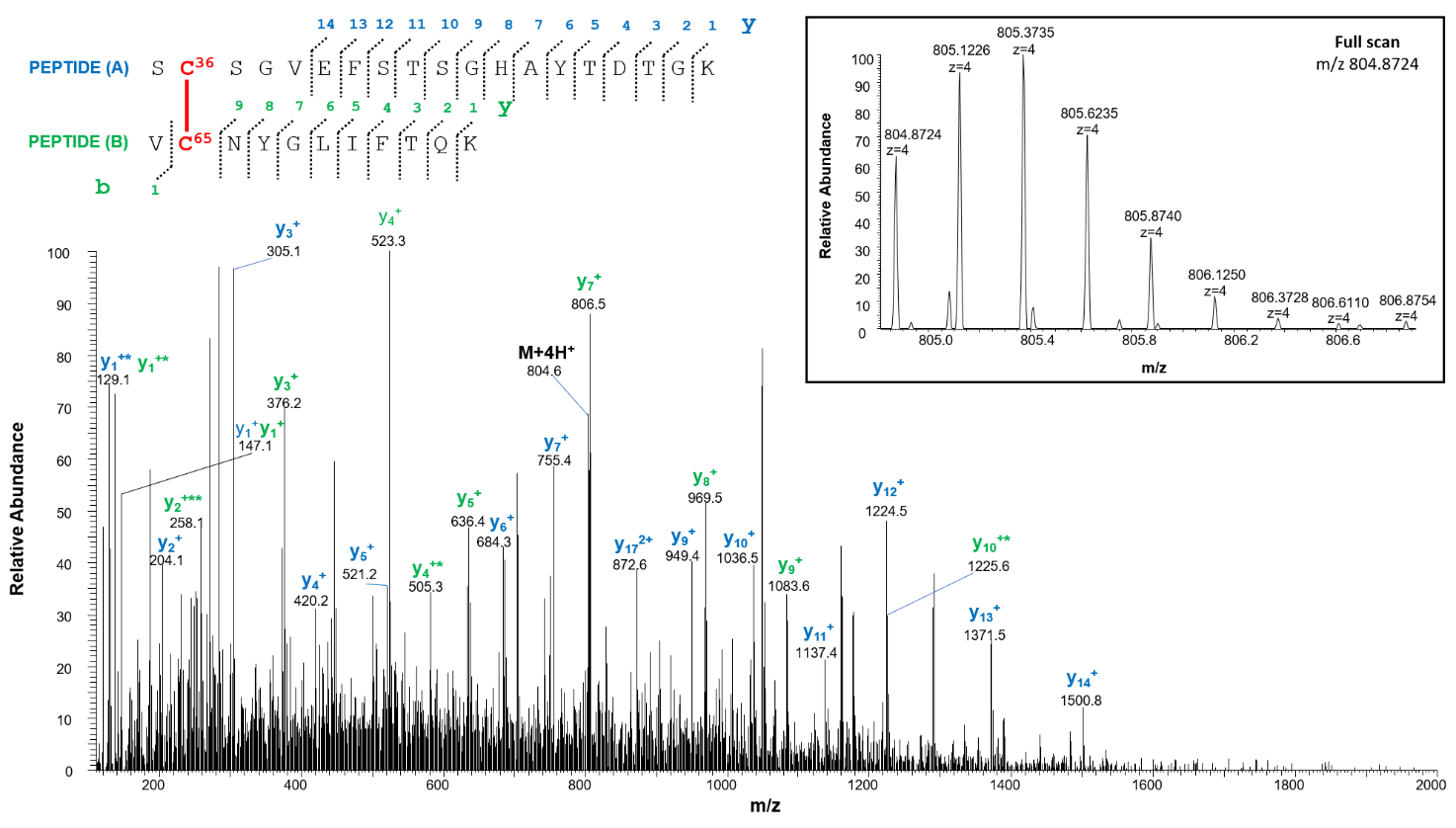


**Figure S4.** MS/MS spectra of the triply charged molecular ion **(A)** at m/z 1072.8252 (calculated 1072.8255) and of the quadruply charged molecular ion **(B)** at m/z 804.8724 (calculated 804.8710) of tryptic peptide 2 (Table 1) of rVDAC3 with cysteine residues 36 and 65 linked by a disulfide bridge. Fragments from peptide A and peptide B are indicated in blue and green, respectively. Fragment ions originated from the neutral loss of H_2_O are indicated by an asterisk. Fragment ions originated from the neutral loss of NH_3_ are indicated by two asterisks. The inset shows the full scan mass spectra of the molecular ions.


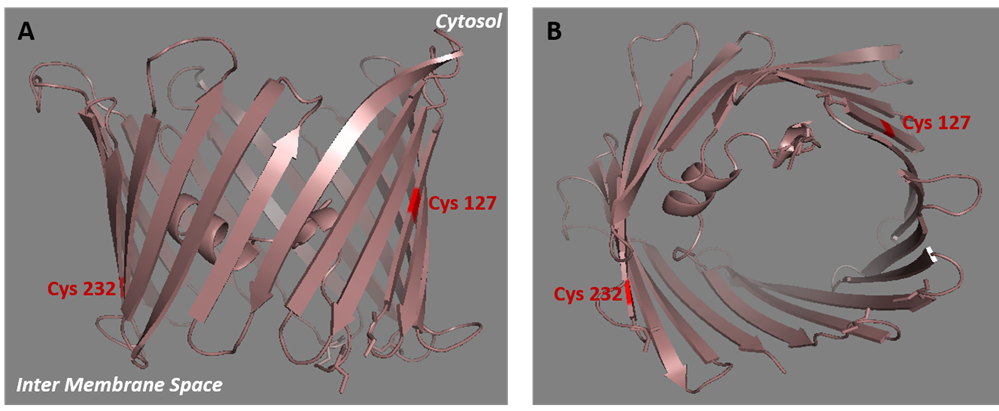


**Figure S5.** Lateral and top view of rVDAC1. The structure of rVDAC1 is predicted by homology modelling, using mouse VDAC1 structure (pdb: 3EMN) as a template. Graphical representation was obtained by using PyMOL 1.1 (DeLano Scientific LLC.). Cysteine residue are shown in red.


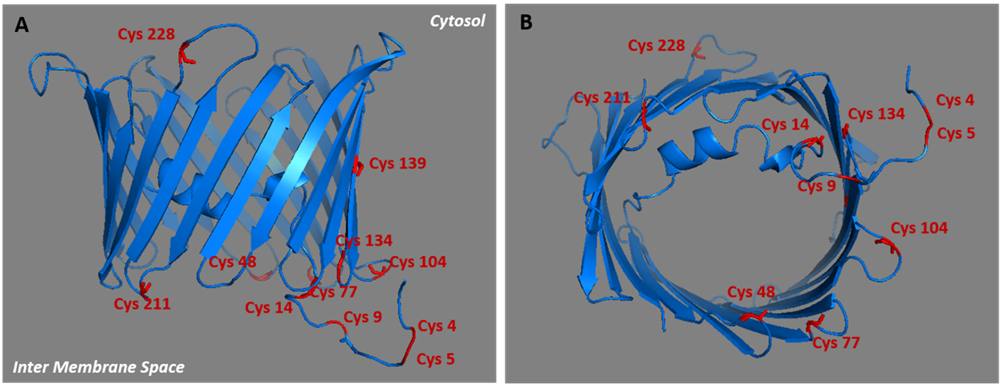


**Figure S6.** Lateral and top view of rVDAC2. The structure of rVDAC2 is predicted by homology modelling, using mouse VDAC1 structure (pdb: 3EMN) as a template. Graphical representation was obtained by using PyMOL 1.1 (DeLano Scientific LLC.). Cysteine residue are shown in red.


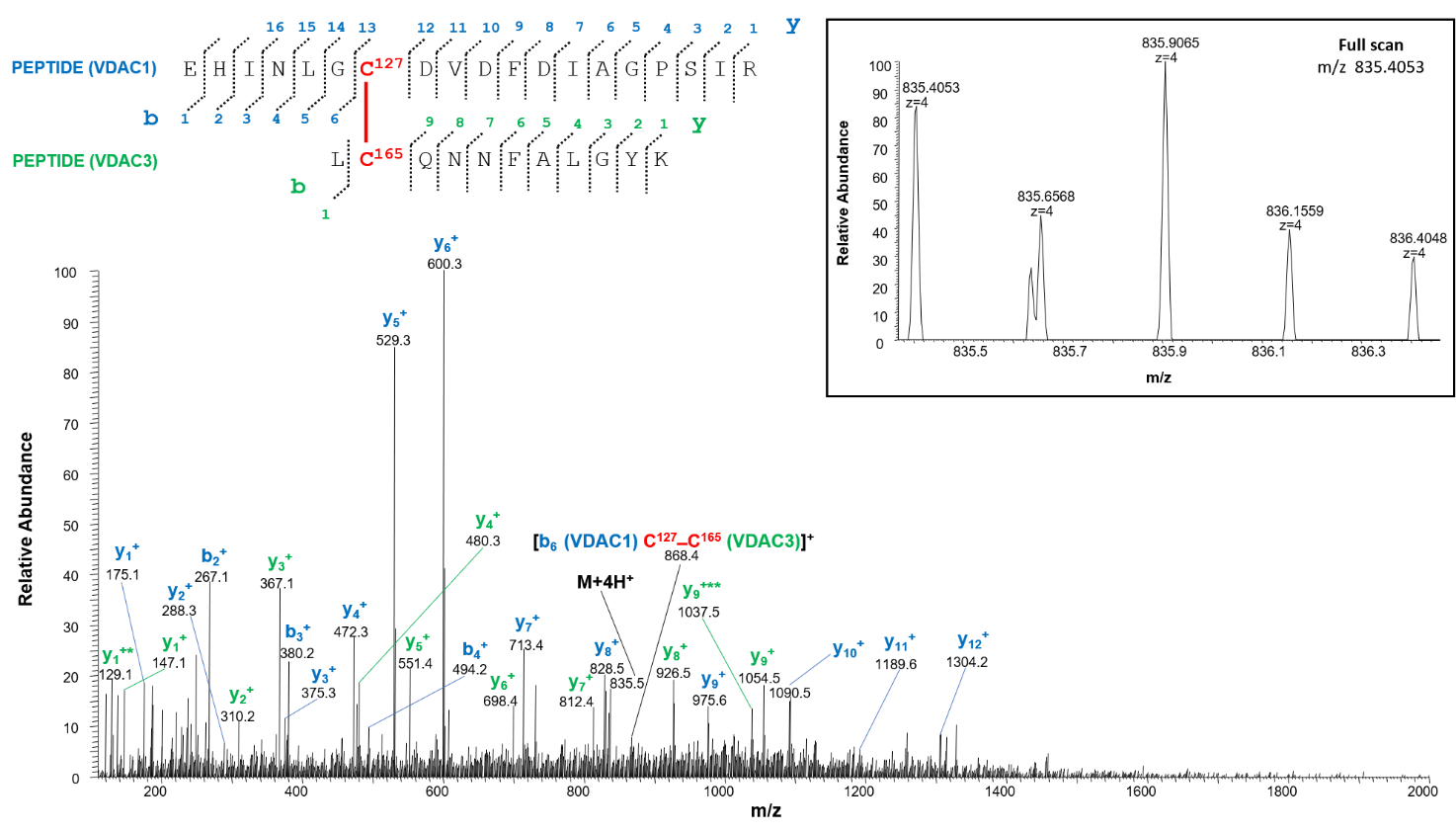


**Figure S7.** MS/MS spectrum of the quadruply charged molecular ion at m/z 835.4053 (calculated 835.4040) of tryptic peptide 2 (Table 3) with cysteine residues 127 of rVDAC1 and 165 of rVDAC3 linked by a disulfide bridge. The fragments with intact disulfide-bond are reported in the MS/MS spectrum. Fragments from peptide (VDAC1) and peptide (VDAC3) are indicated in blue and green, respectively. Fragment ions originated from the neutral loss of H_2_O are indicated by an asterisk. Fragment ions originated from the neutral loss of NH_3_ are indicated by two asterisks. The inset shows the full scan mass spectrum of the molecular ion.


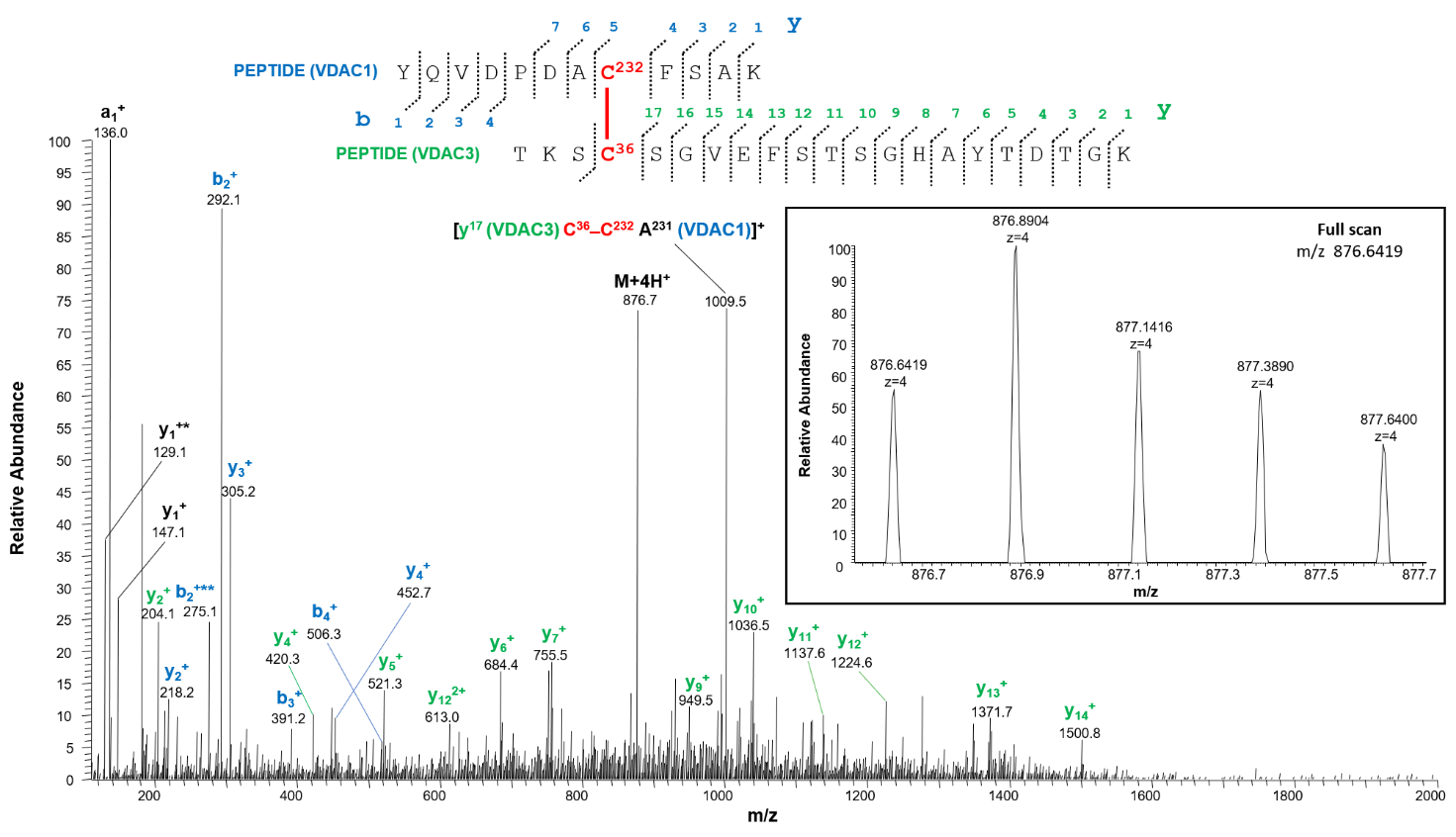


**Figure S8.** MS/MS spectrum of the quadruply charged molecular ion at m/z 876.6419 (calculated 876.6402) of tryptic peptide 3 (Table 3) with cysteine residues 232 of rVDAC1 and 36 of rVDAC3 linked by a disulfide bridge. The fragments with intact disulfide-bond are reported in the MS/MS spectrum. Fragments from peptide (VDAC1) and peptide (VDAC3) are indicated in blue and green, respectively. Fragment ions originated from the neutral loss of H_2_O are indicated by an asterisk. Fragment ions originated from the neutral loss of NH_3_ are indicated by two asterisks. The inset shows the full scan mass spectrum of the molecular ion.


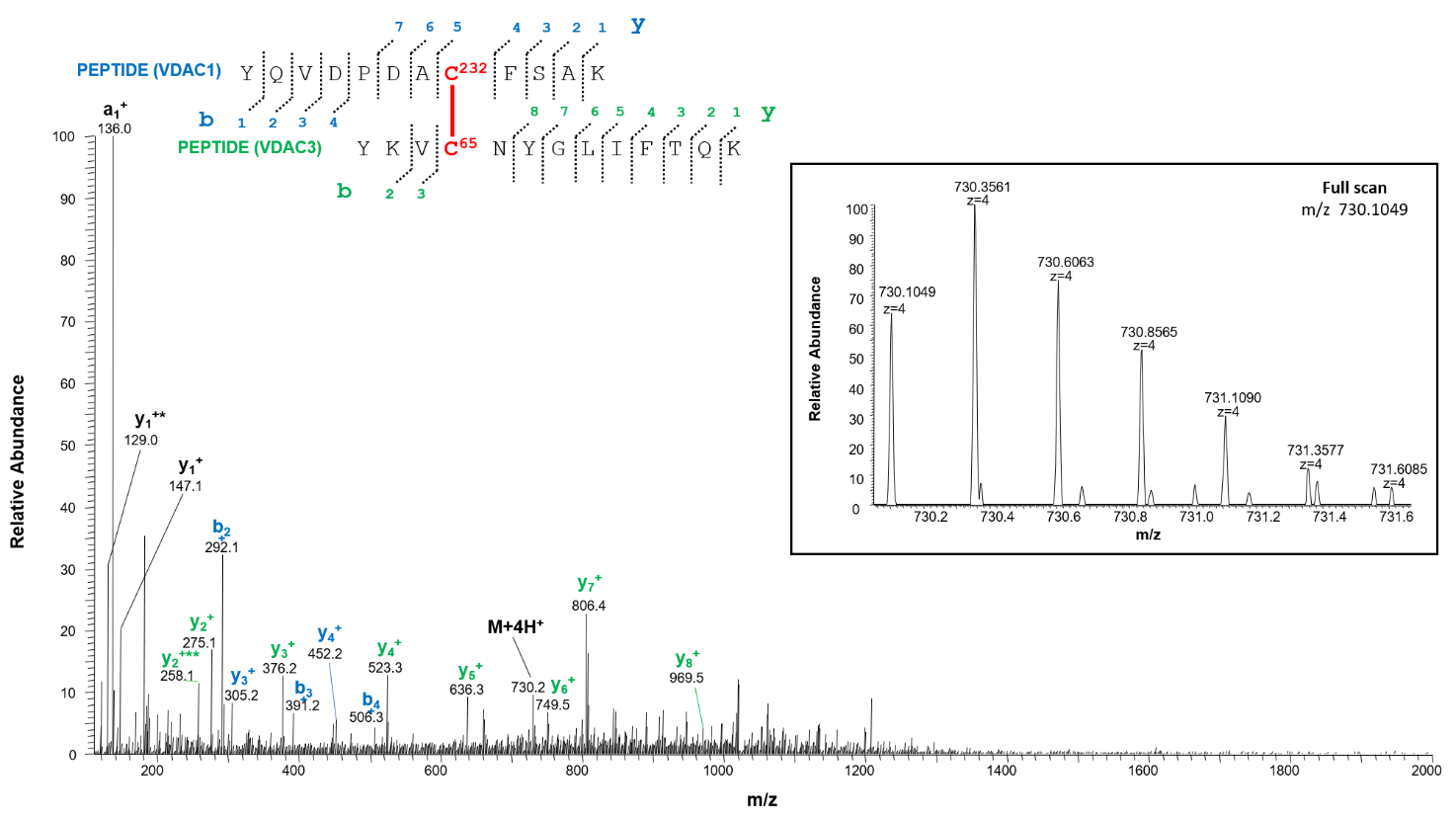


**Figure S9.** MS/MS spectrum of the quadruply charged molecular ion at m/z 730.1049 (calculated 730.1032) of tryptic peptide 4 (Table 3) with cysteine residues 232 of rVDAC1 and 65 of rVDAC3 linked by a disulfide bridge. Fragments from peptide (VDAC1) and peptide (VDAC3) are indicated in blue and green, respectively. Fragment ions originated from the neutral loss of H_2_O are indicated by an asterisk. Fragment ions originated from the neutral loss of NH_3_ are indicated by two asterisks. The inset shows the full scan mass spectrum of the molecular ion.


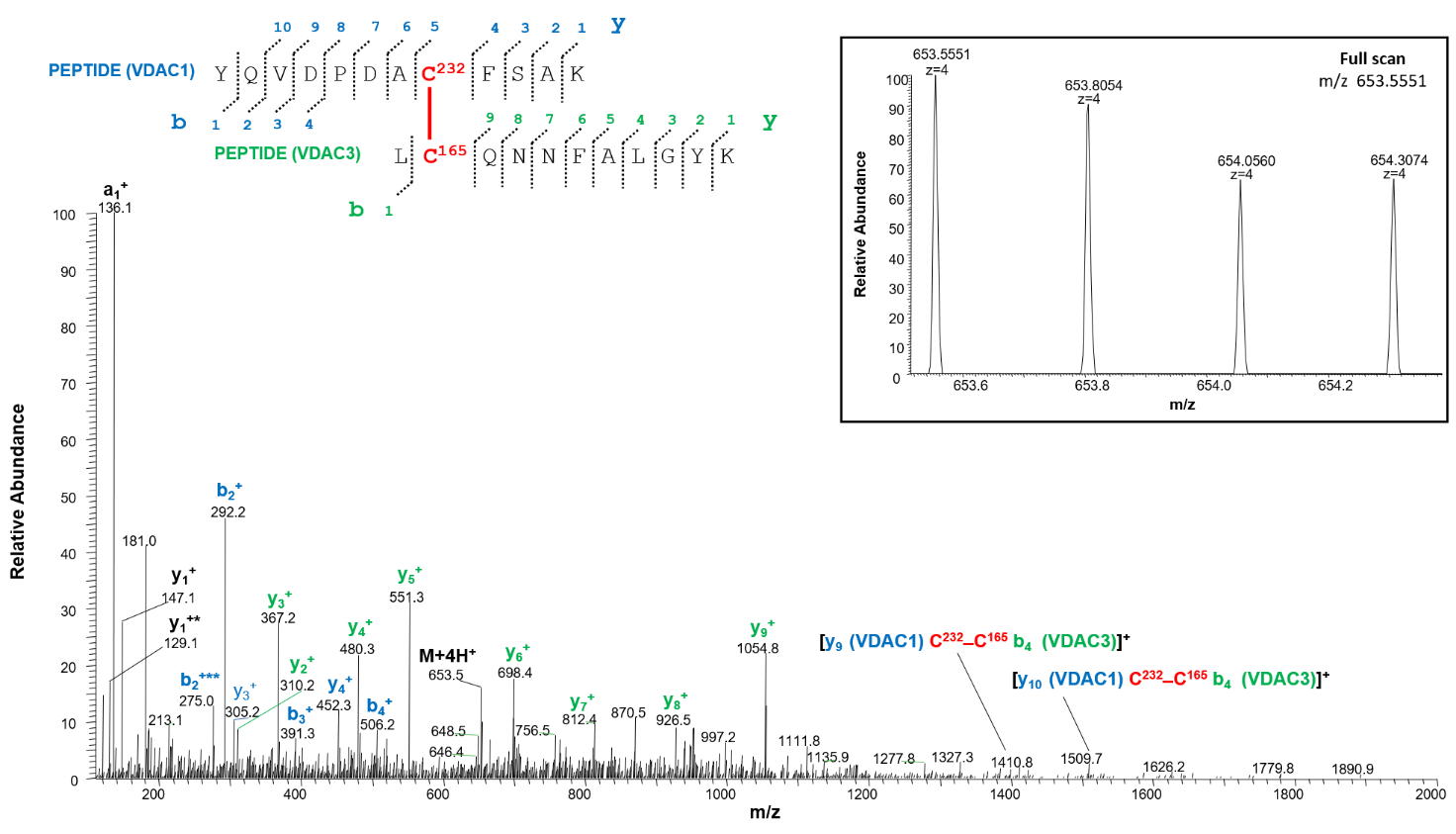


**Figure S10.** MS/MS spectrum of the quadruply charged molecular ion at m/z 653.5551 (calculated 653.5546) of tryptic peptide 5 (Table 3) with cysteine residues 232 of rVDAC1 and 165 of rVDAC3 linked by a disulfide bridge. The fragments with intact disulfide-bond are reported in the MS/MS spectrum. Fragments from peptide (VDAC1) and peptide (VDAC3) are indicated in blue and green, respectively. Fragment ions originated from the neutral loss of H_2_O are indicated by an asterisk. Fragment ions originated from the neutral loss of NH_3_ are indicated by two asterisks. The inset shows the full scan mass spectrum of the molecular ion.


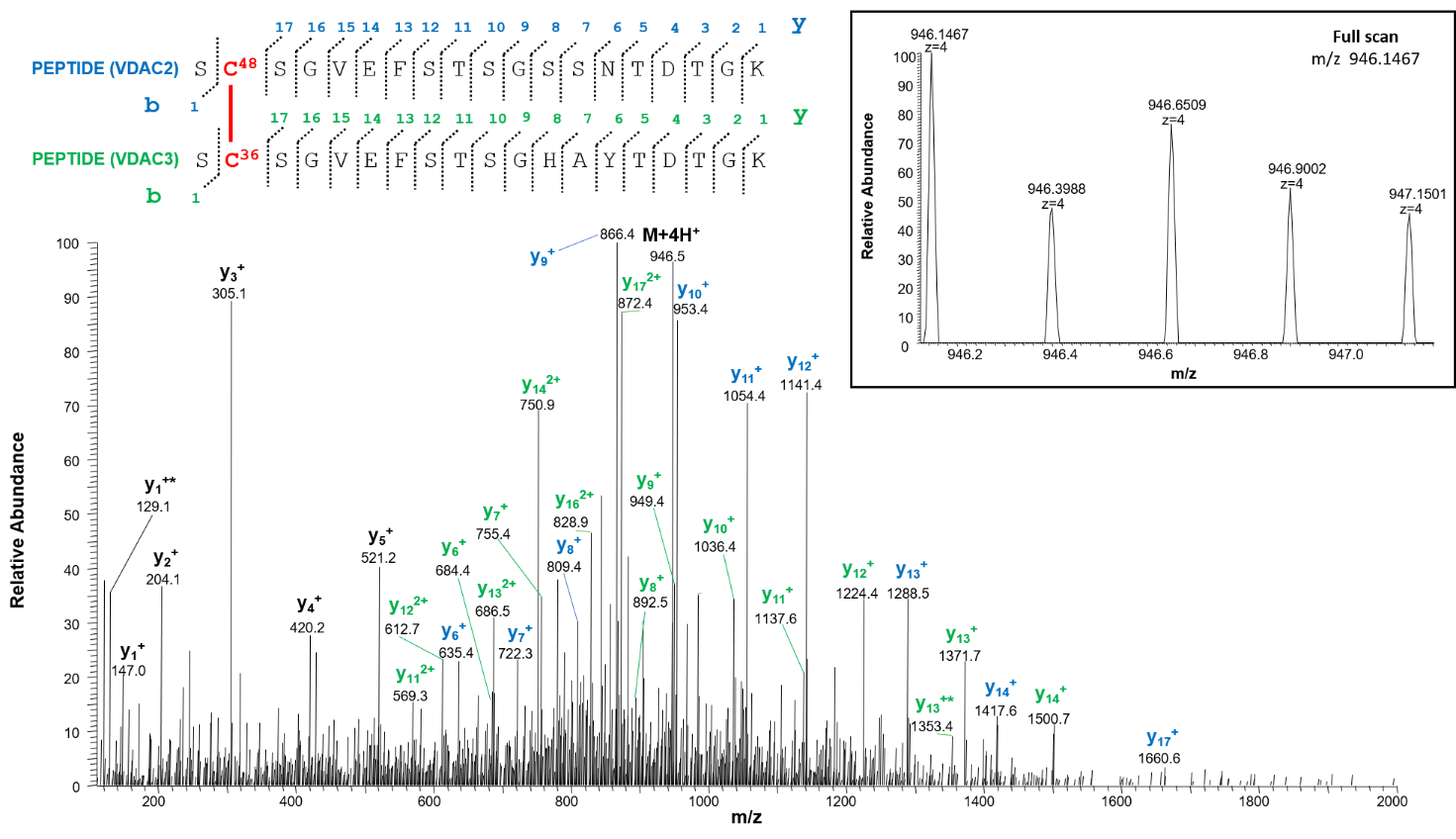


**Figure S11.** MS/MS spectrum of the quadruply charged molecular ion at m/z 946.1467 (calculated 946.1485) of tryptic peptide 6 (Table 3) with cysteine residues 48 of rVDAC2 and 36 of rVDAC3 linked by a disulfide bridge. The fragments with intact disulfide-bond are reported in the MS/MS spectrum. Fragments from peptide (VDAC2) and peptide (VDAC3) are indicated in blue and green, respectively. Fragment ions originated from the neutral loss of H_2_O are indicated by an asterisk. Fragment ions originated from the neutral loss of NH_3_ are indicated by two asterisks. The inset shows the full scan mass spectrum of the molecular ion.


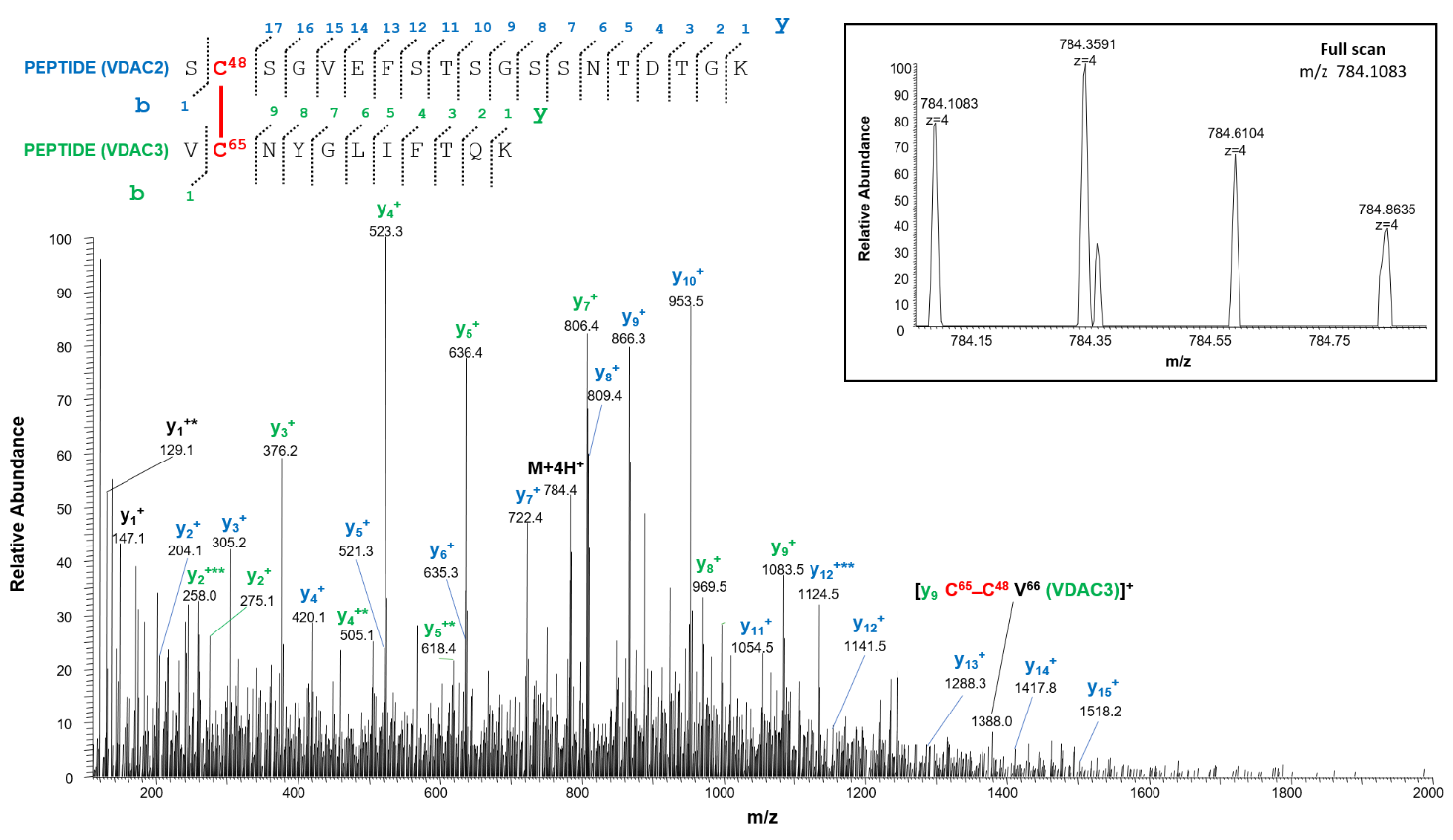


**Figure S12.** MS/MS spectrum of the quadruply charged molecular ion at m/z 784.1083 (calculated 784.1080) of tryptic peptide 7 (Table 3) with cysteine residues 48 of rVDAC2 and 65 of rVDAC3 linked by a disulfide bridge. The fragments with intact disulfide-bond are reported in the MS/MS spectrum. Fragments from peptide (VDAC2) and peptide (VDAC3) are indicated in blue and green, respectively. Fragment ions originated from the neutral loss of H_2_O are indicated by an asterisk. Fragment ions originated from the neutral loss of NH_3_ are indicated by two asterisks. The inset shows the full scan mass spectrum of the molecular ion.

**A)**


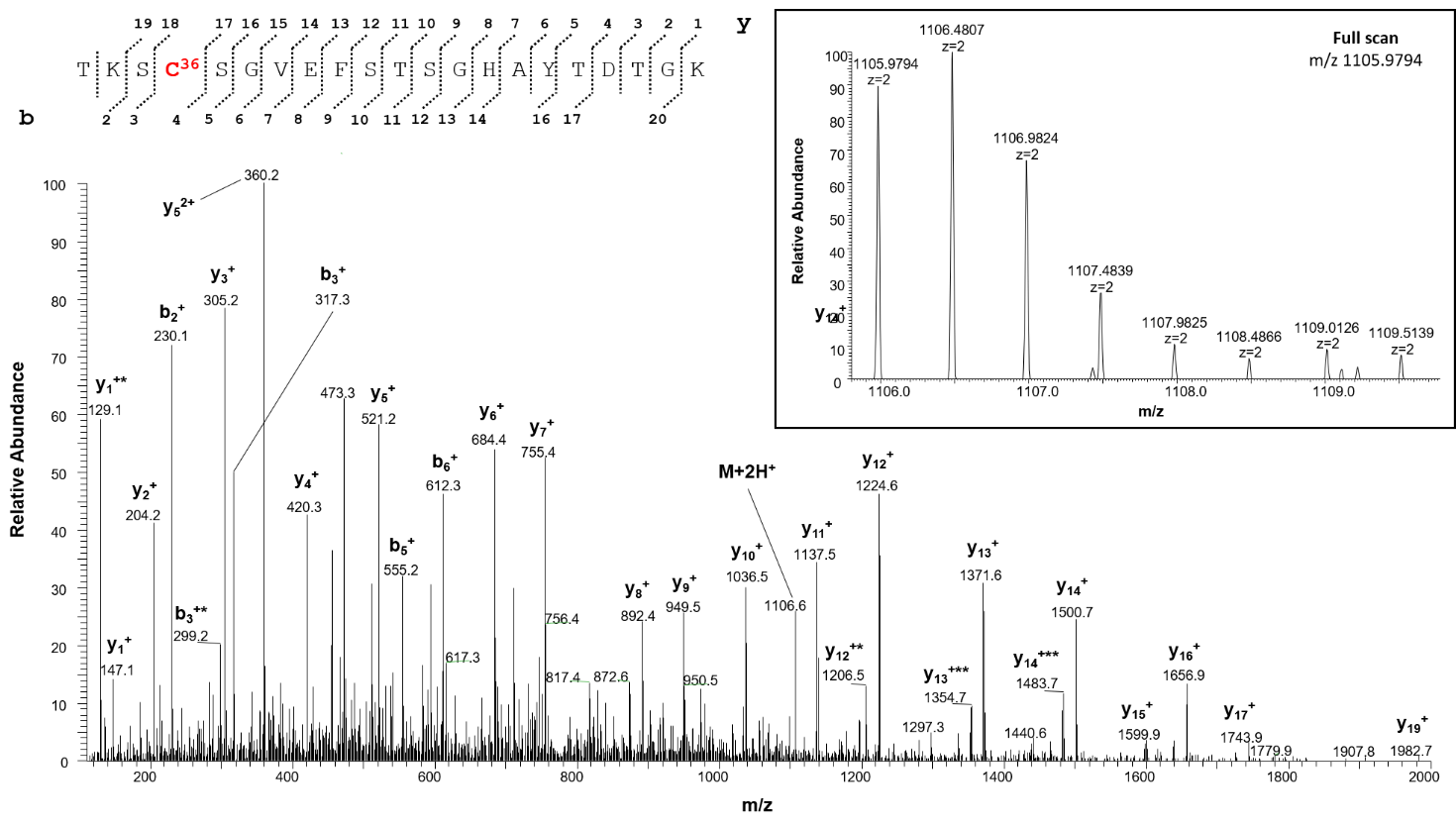


**B)**


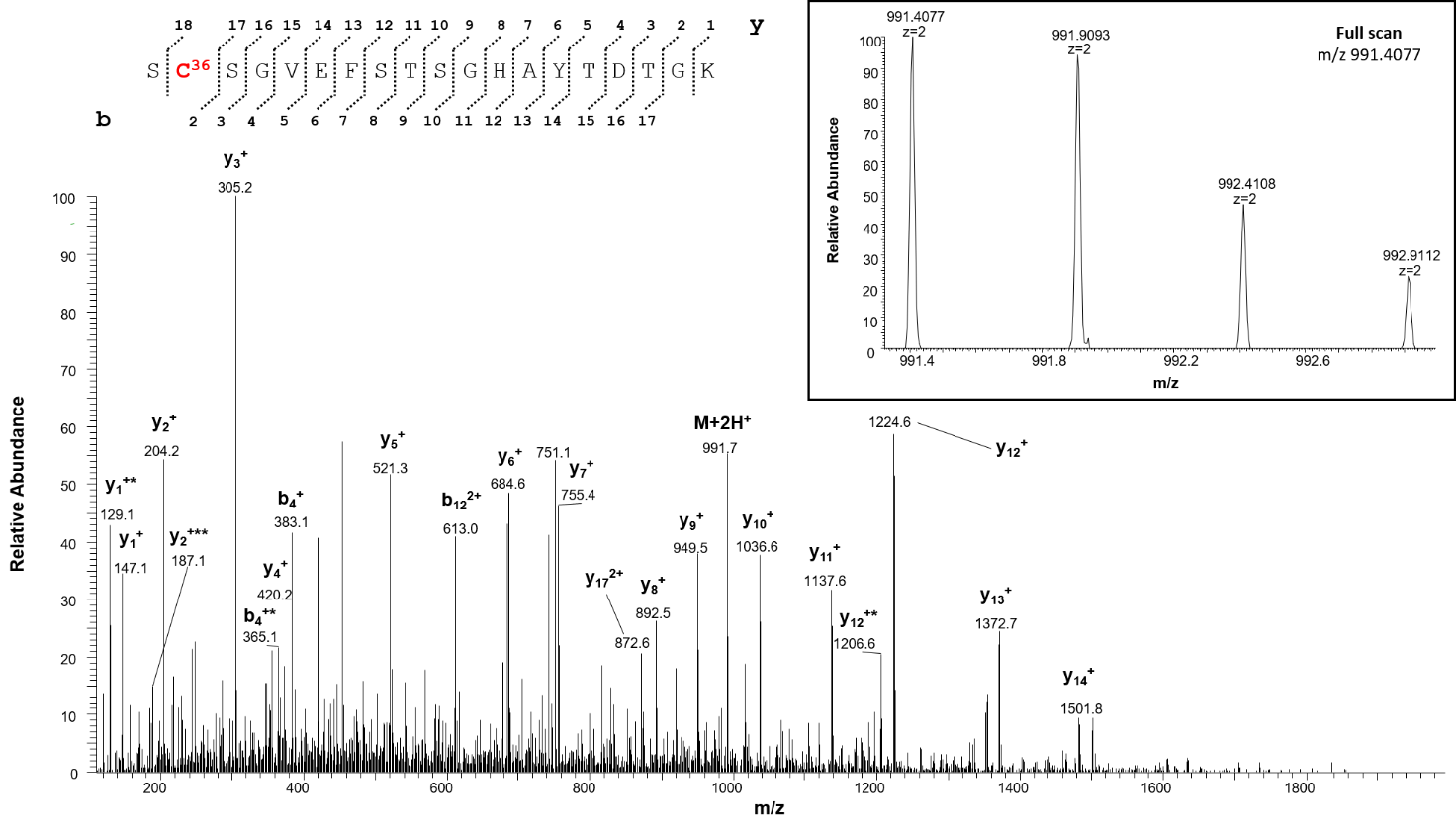


**Figure S13.** MS/MS spectrum of the doubly charged molecular ions at m/z 1105.9794 **(A)** (calculated 1105.9792) and 991.4077 **(B)** (calculated 991.4079) of tryptic peptides 1 and 2 (Table S1) of rVDAC3 with the cysteine residue 36 in the oxidized form to sulfonic acid. Fragment ions originated from the neutral loss of H_2_O are indicated by an asterisk. Fragment ions originated from the neutral loss of NH_3_ are indicated by two asterisks. The inset shows the full scan mass spectrum of molecular ion.

**A)**


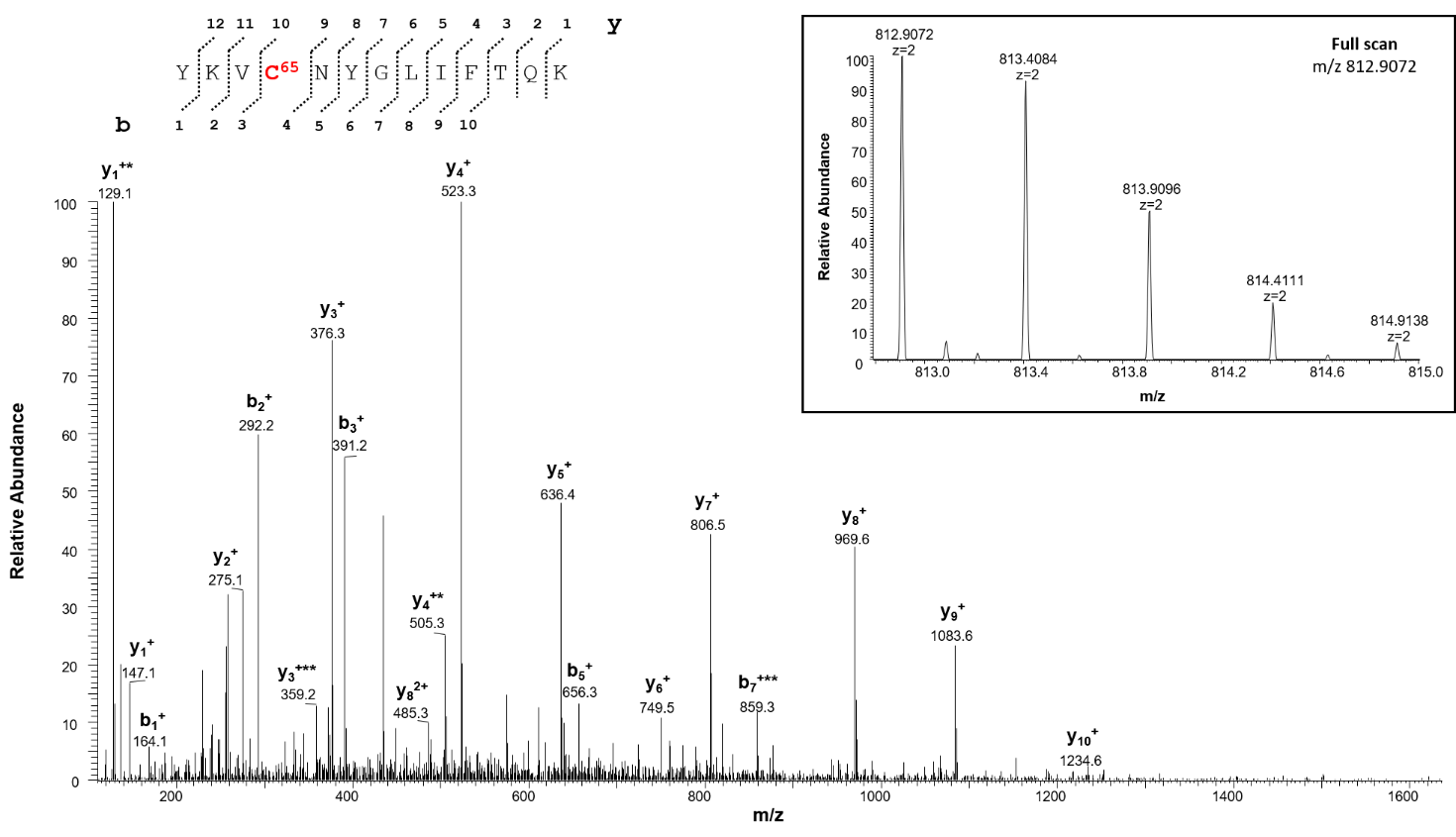


**B)**


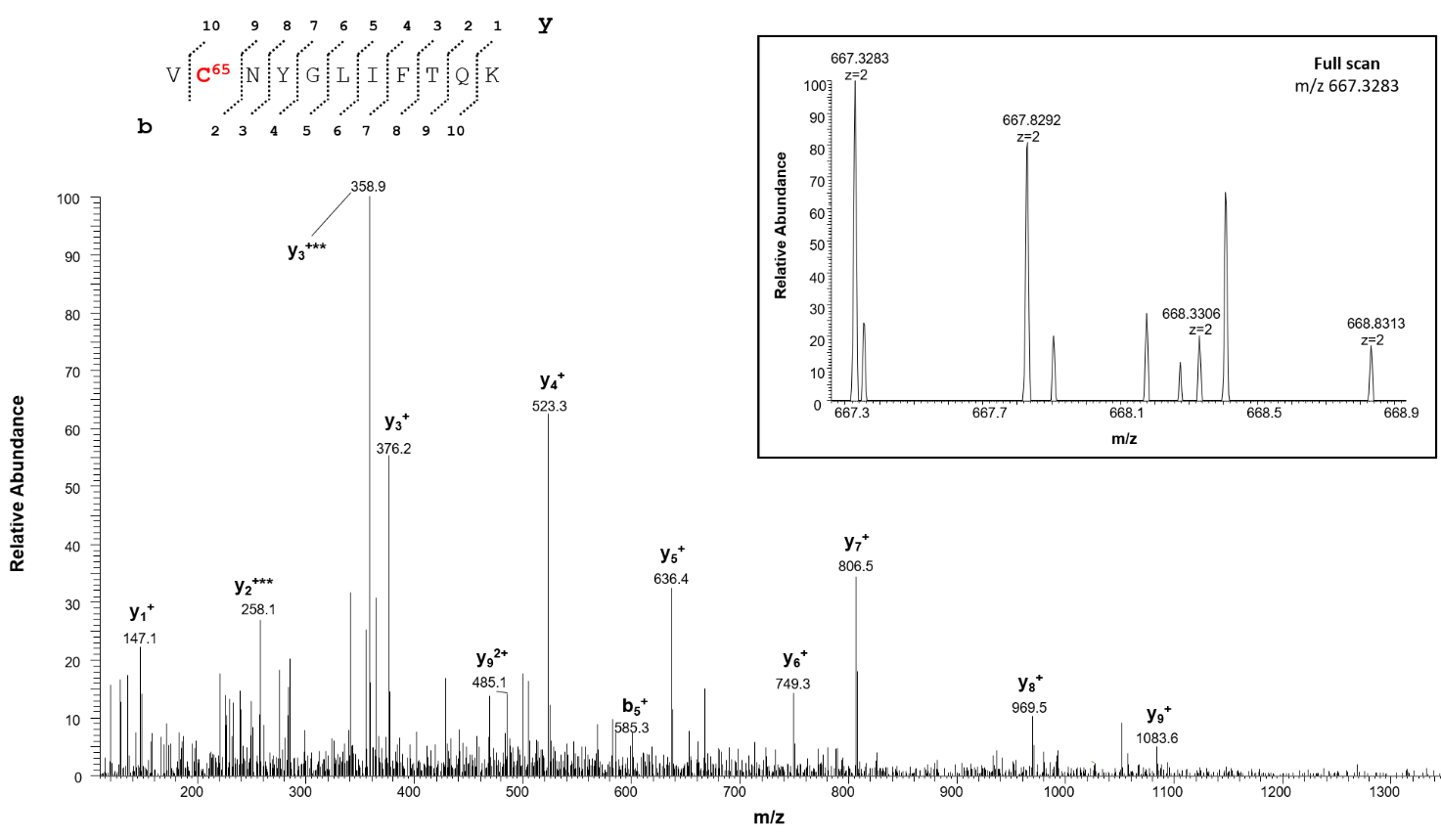


**Figure S14.** MS/MS spectrum of the doubly charged molecular ions at m/z 812.9072 **(A)** (calculated 812.9059) and 667.3283 **(B)** (calculated 667.3267) of tryptic peptides 3 and 4 (Table S1) of rVDAC3 with the cysteine residue 65 in the oxidized form to sulfonic acid. Fragment ions originated from the neutral loss of H_2_O are indicated by an asterisk. Fragment ions originated from the neutral loss of NH_3_ are indicated by two asterisks. The inset shows the full scan mass spectrum of molecular ion.

**A)**


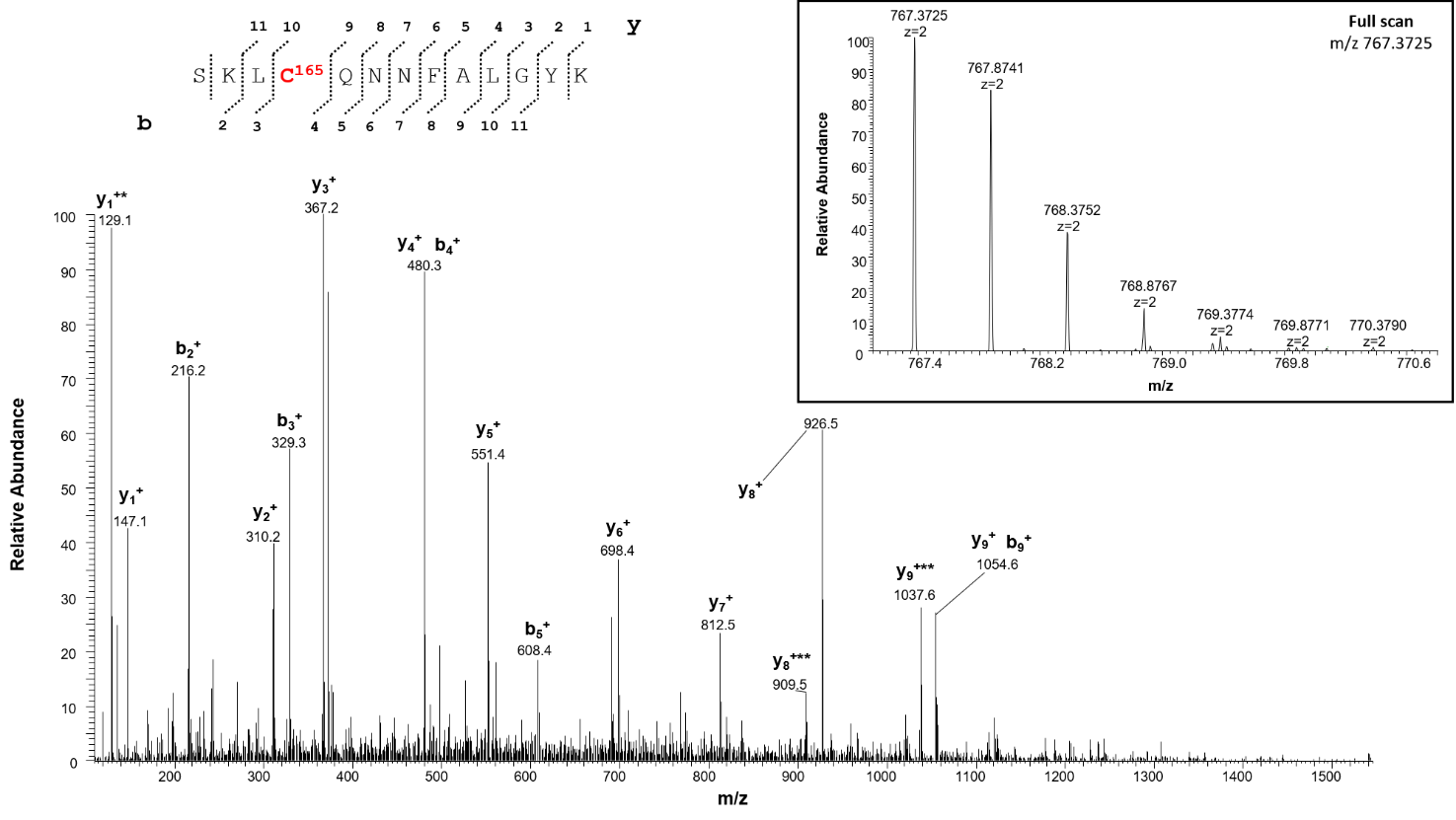


**B)**


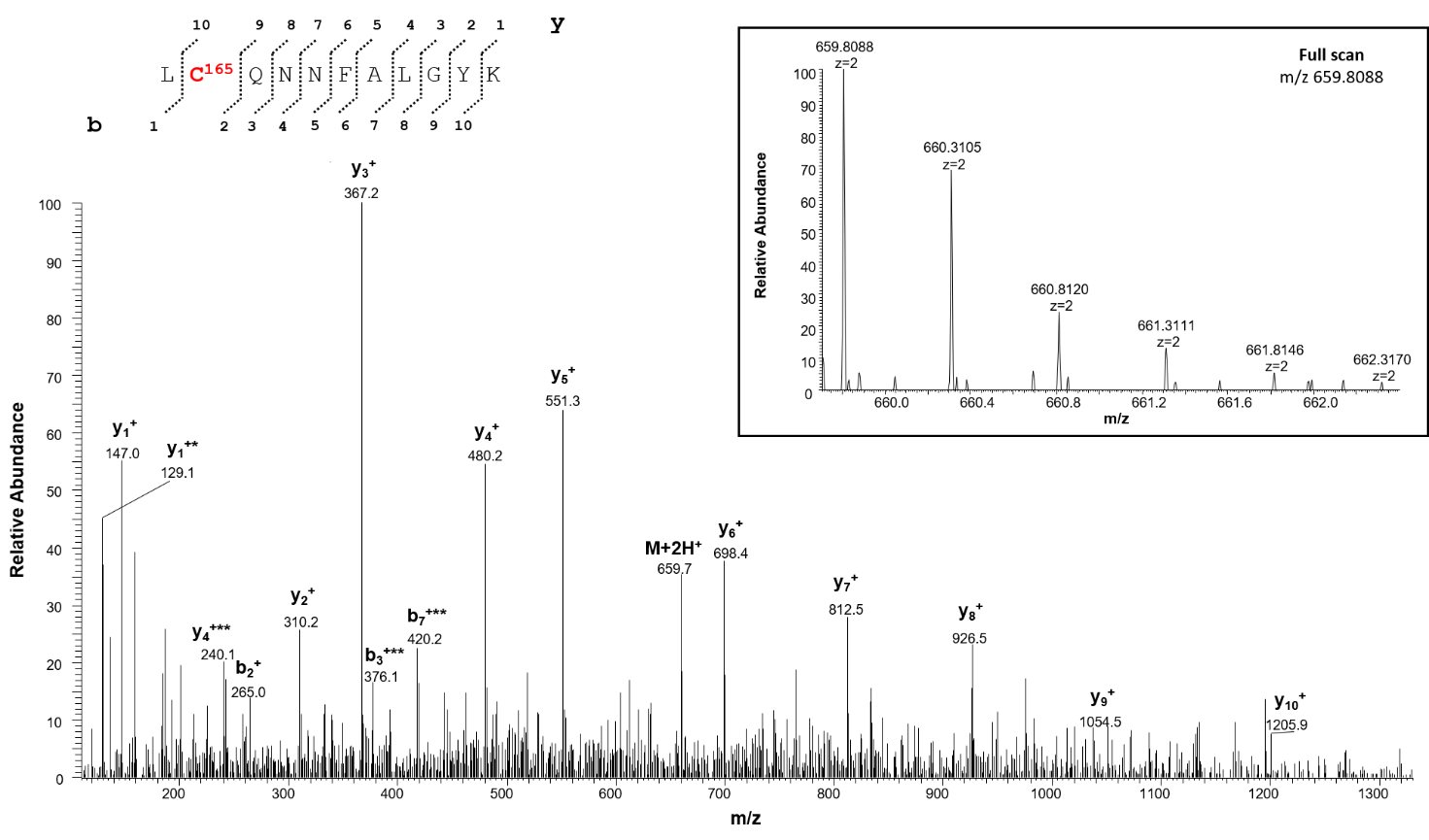


**Figure S15.** MS/MS spectrum of the doubly charged molecular ions at m/z 767.3725 **(A)** (calculated 767.3722) and 659.8088 **(B)** (calculated 659.8087) of tryptic peptides 5 and 6 (Table S1) of rVDAC3 with the cysteine residue 165 in the oxidized form to sulfonic acid. Fragment ions originated from the neutral loss of H_2_O are indicated by an asterisk. Fragment ions originated from the neutral loss of NH_3_ are indicated by two asterisks. The inset shows the full scan mass spectrum of molecular ion.


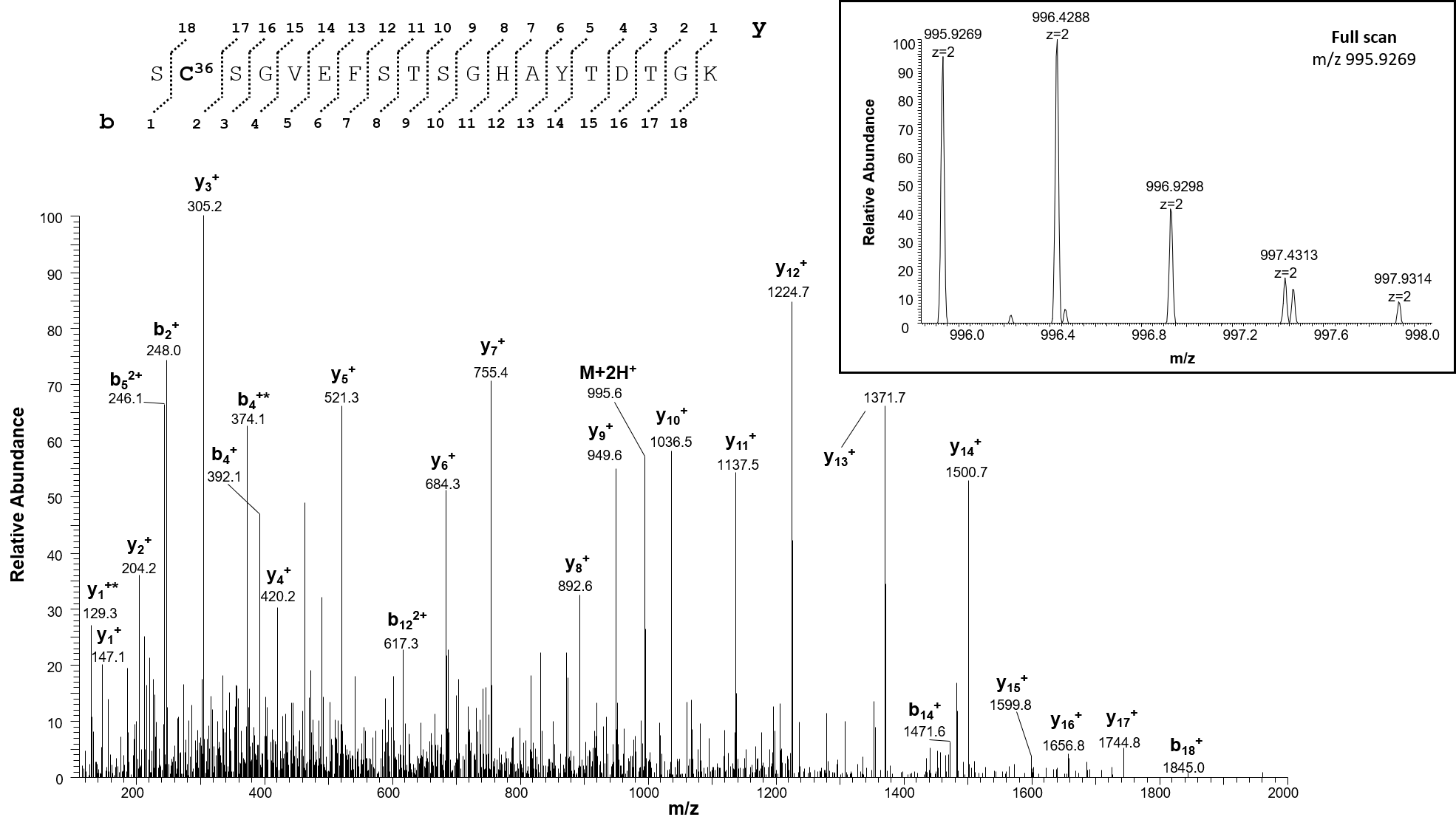


**Figure S16.** MS/MS spectrum of the doubly charged molecular ion at m/z 995.9269 (calculated 995.9260) of tryptic peptide 2 (Table 2) of rVDAC3 with the cysteine residue 36 in the carboxyamidomethylated form. Fragment ions originated from the neutral loss of H_2_O are indicated by an asterisk. Fragment ions originated from the neutral loss of NH_3_ are indicated by two asterisks. The inset shows the full scan mass spectrum of molecular ion.

**A)**
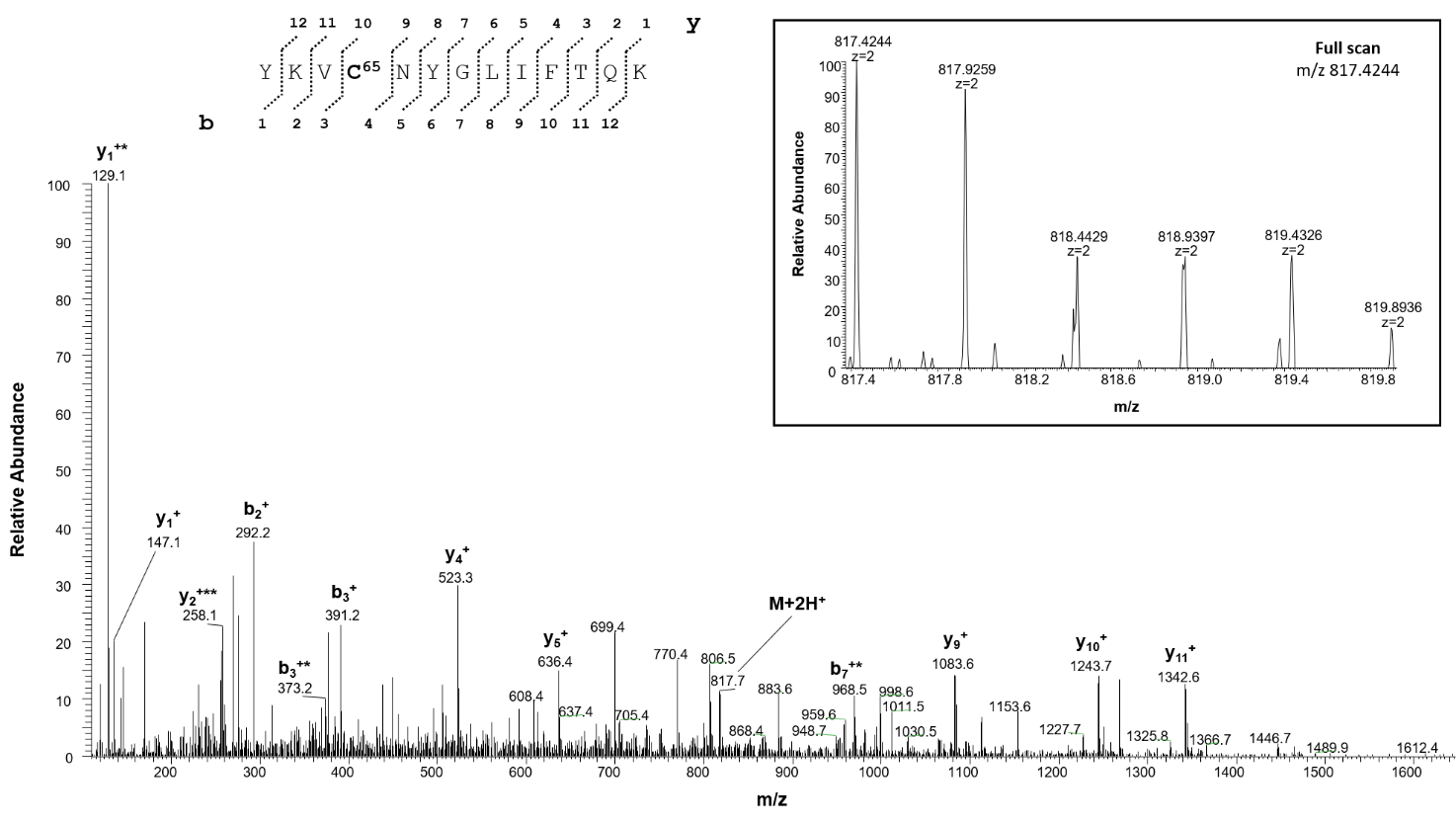


**B)**


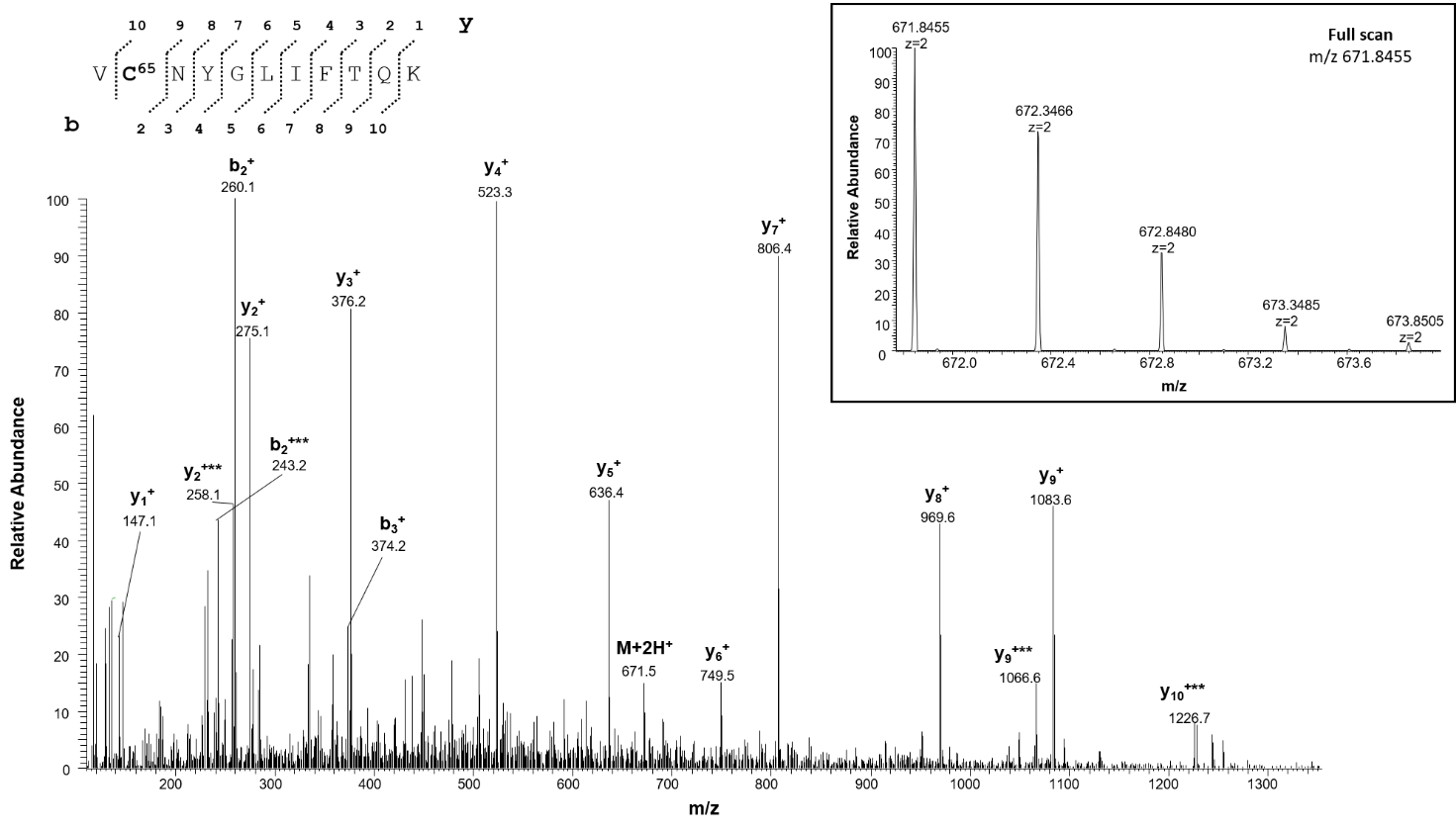


**Figure S17.** MS/MS spectrum of the doubly charged molecular ions at m/z 817.4244 **(A)** (calculated 817.4240) and 671.8455 **(B)** (calculated 671.8448) of tryptic peptides 3 and 4 (Table S2) of rVDAC3 with the cysteine residue 65 in the carboxyamidomethylated form. Fragment ions originated from the neutral loss of H_2_O are indicated by an asterisk. Fragment ions originated from the neutral loss of NH_3_ are indicated by two asterisks. The inset shows the full scan mass spectrum of molecular ion.

**A)**


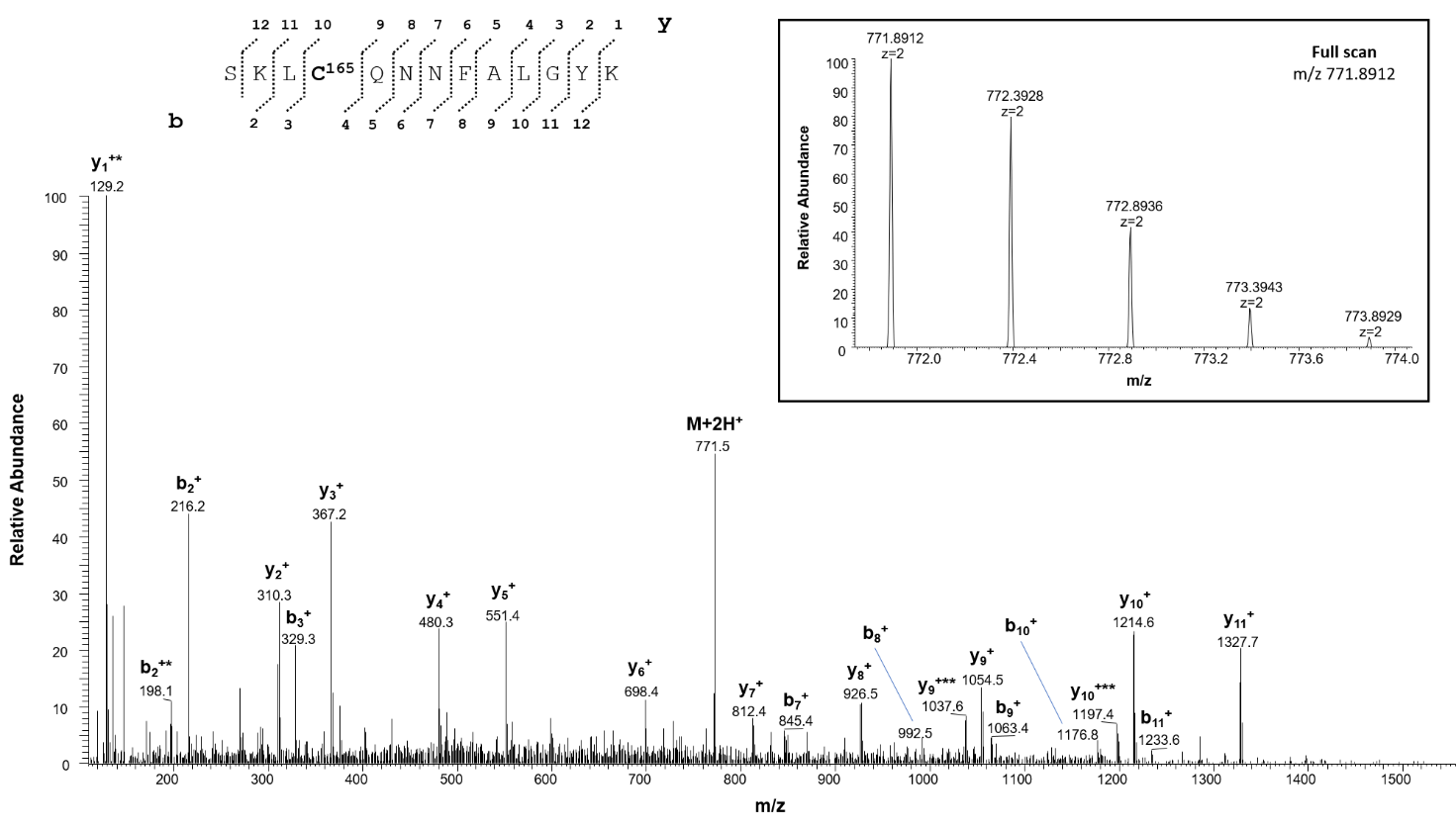


**B)**


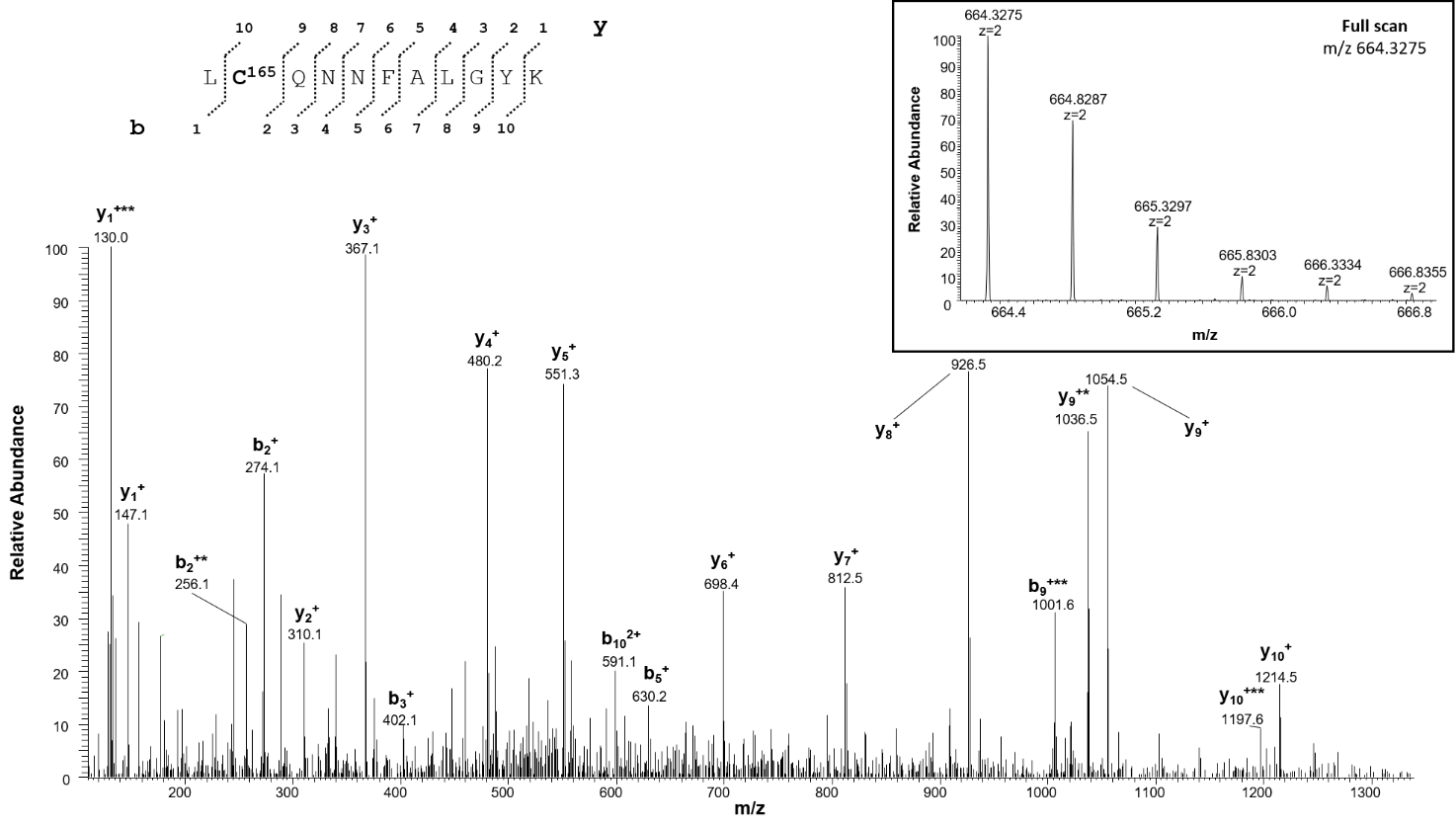


**Figure S18.** MS/MS spectrum of the doubly charged molecular ions at m/z 771.8912 **(A)** (calculated 771.8903) and 664.3275 **(B)** (calculated 664.3268) of tryptic peptides 6 and 7 (Table S2) of rVDAC3 with the cysteine residue 165 in the carboxyamidomethylated form. Fragment ions originated from the neutral loss of H_2_O are indicated by an asterisk. Fragment ions originated from the neutral loss of NH_3_ are indicated by two asterisks. The inset shows the full scan mass spectrum of molecular ion.


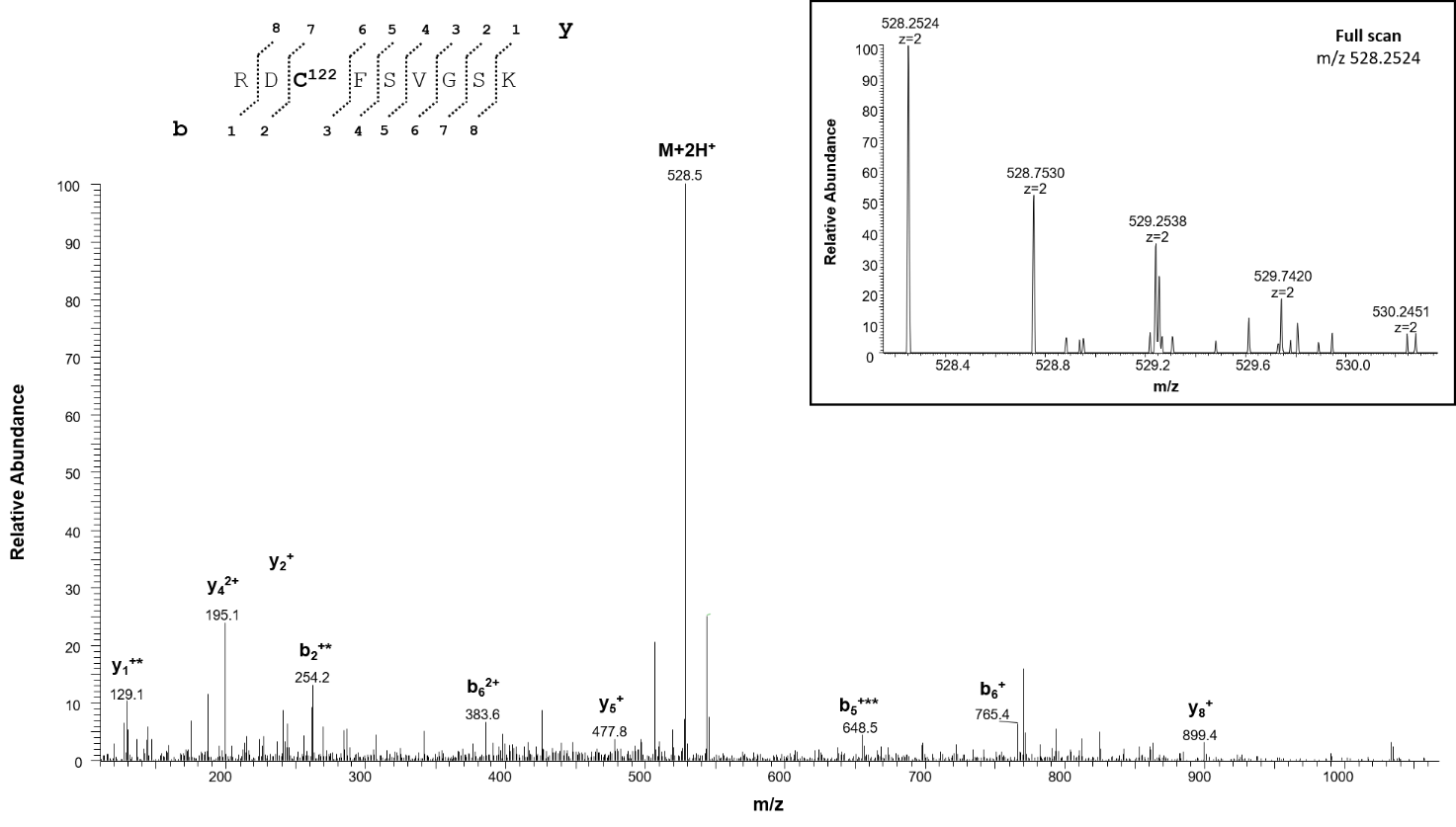


**Figure S19.** MS/MS spectrum of the doubly charged molecular ion at m/z 528.2524 (calculated 528.2511) of tryptic peptide 5 (Table S2) of rVDAC3 with the cysteine residue 122 in the carboxyamidomethylated form. Fragment ions originated from the neutral loss of H_2_O are indicated by an asterisk. Fragment ions originated from the neutral loss of NH_3_ are indicated by two asterisks. The inset shows the full scan mass spectrum of molecular ion.


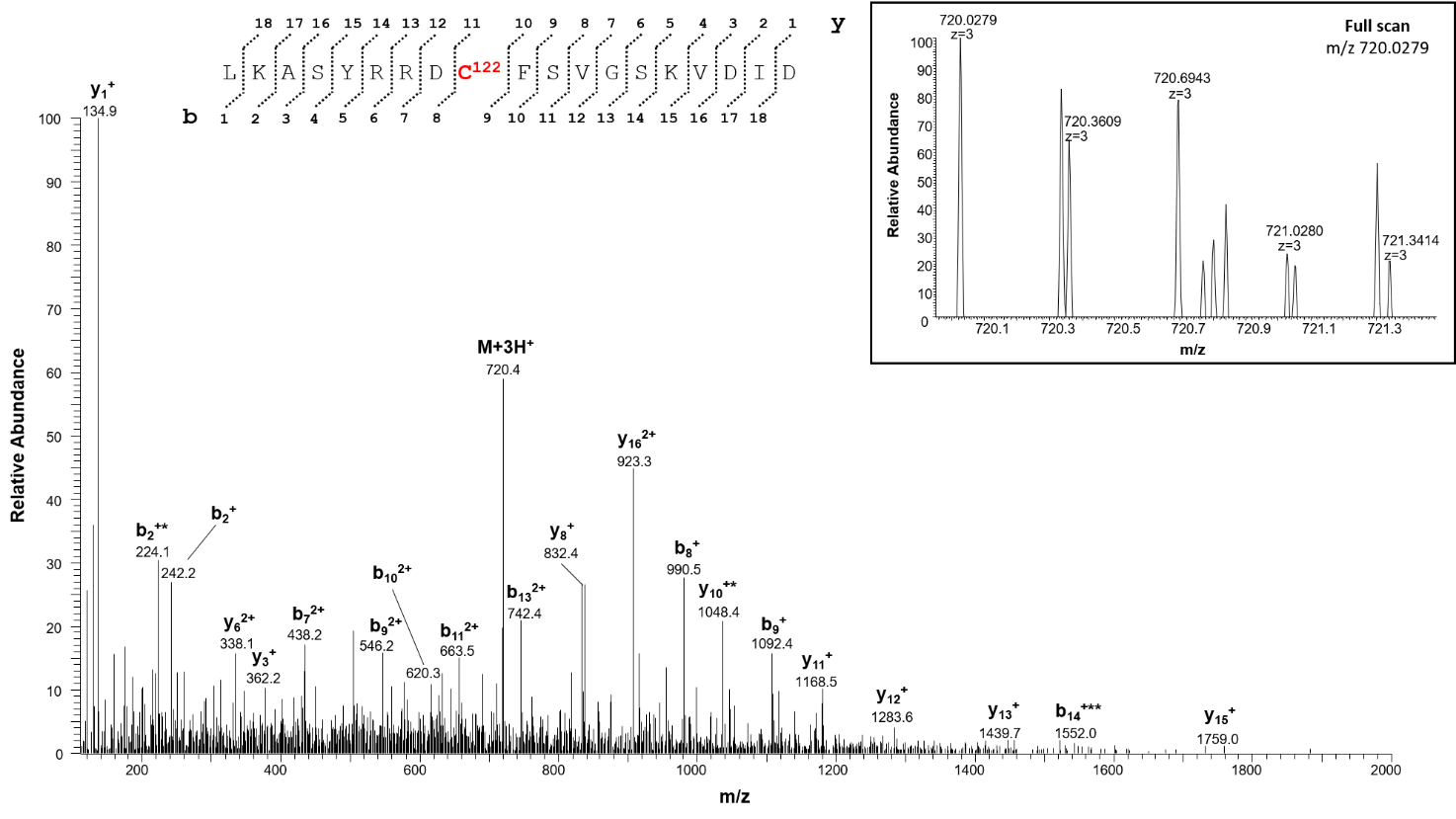


**Figure S20.** MS/MS spectrum of the triply charged molecular ion at m/z 720.0279 (calculated 720.0332) of the non-specific tryptic peptide 1 (Table S6) of rVDAC3 with the cysteine residue 122 with the variable modification “Half of a disulfide bridge”. Fragment ions originated from the neutral loss of H2O are indicated by an asterisk. Fragment ions originated from the neutral loss of NH3 are indicated by two asterisks. The inset shows the full scan mass spectrum of molecular ion.


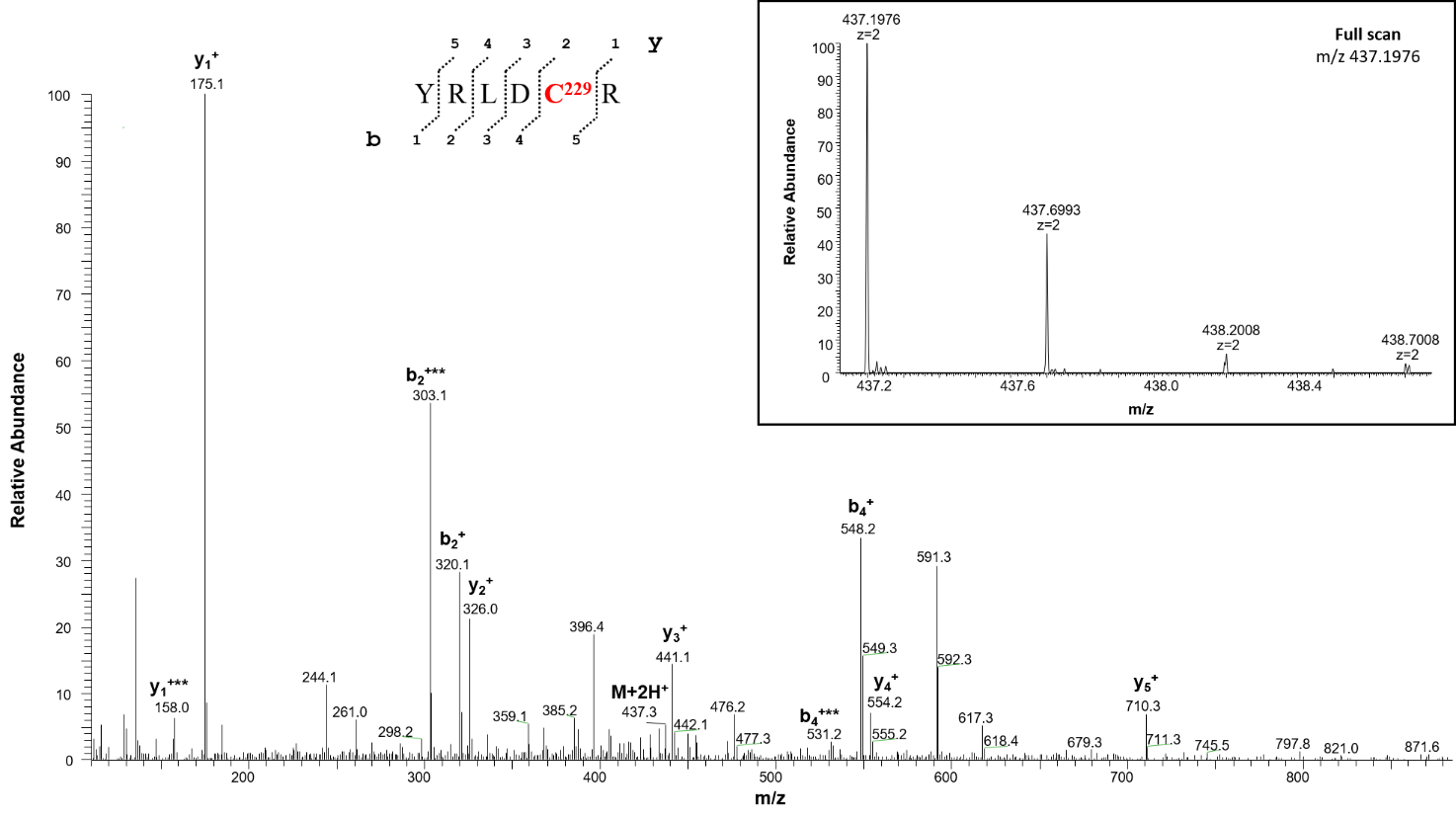


**Figure S21.** MS/MS spectrum of the doubly charged molecular ion at m/z 437.1976 (calculated 437.1983) of tryptic peptide 7 (Table S1) of rVDAC3 with the cysteine residue 229 in the oxidized form to sulfonic acid. Fragment ions originated from the neutral loss of H_2_O are indicated by an asterisk. Fragment ions originated from the neutral loss of NH_3_ are indicated by two asterisks. The inset shows the full scan mass spectrum of molecular ion.


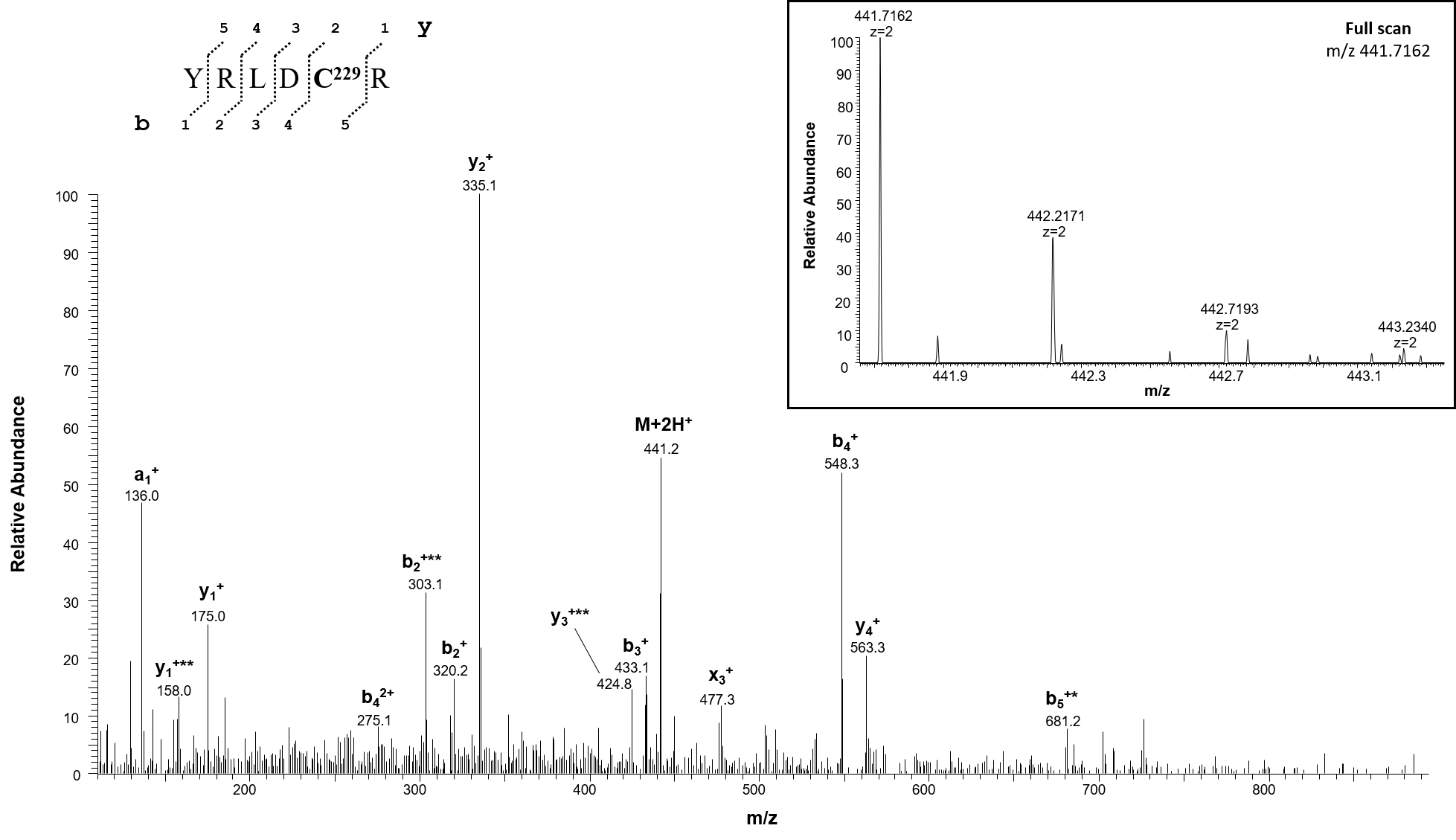


**Figure S22.** MS/MS spectrum of the doubly charged molecular ion at m/z 441.7162 (calculated 441.7167) of tryptic peptide 8 (Table S2) of rVDAC3 with the cysteine residue 229 in the carboxyamidomethylated form. Fragment ions originated from the neutral loss of H_2_O are indicated by an asterisk. Fragment ions originated from the neutral loss of NH_3_ are indicated by two asterisks. The inset shows the full scan mass spectrum of molecular ion.
